# Regulation of RNA editing by RNA-binding proteins in human cells

**DOI:** 10.1038/s42003-018-0271-8

**Published:** 2019-01-14

**Authors:** Giovanni Quinones-Valdez, Stephen S. Tran, Hyun-Ik Jun, Jae Hoon Bahn, Ei-Wen Yang, Lijun Zhan, Anneke Brümmer, Xintao Wei, Eric L. Van Nostrand, Gabriel A. Pratt, Gene W. Yeo, Brenton R. Graveley, Xinshu Xiao

**Affiliations:** 1 Department of Bioengineering, University of California Los Angeles, Los Angeles, CA 90095 USA; 2 Bioinformatics Interdepartmental Program, University of California Los Angeles, Los Angeles, CA 90095 USA; 3 Department of Integrative Biology and Physiology, University of California Los Angeles, Los Angeles, CA 90095 USA; 4 Department of Genetics and Genome Sciences, Institute for Systems Genomics, UConn Health, Farmington, CT 06030 USA; 5 Department of Cellular and Molecular Medicine, University of California San Diego, La Jolla, CA 92093 USA; 6 Institute for Genomic Medicine, University of California San Diego, La Jolla, CA 92093 USA; 7 Bioinformatics and Systems Biology Graduate Program, University of California San Diego, La Jolla, CA 92093 USA; 8 Molecular Biology Institute, University of California Los Angeles, Los Angeles, CA 90095 USA; 9 Institute for Quantitative and Computational Biology, University of California Los Angeles, Los Angeles, CA 90095 USA

## Abstract

Adenosine-to-inosine (A-to-I) editing, mediated by the ADAR enzymes, diversifies the transcriptome by altering RNA sequences. Recent studies reported global changes in RNA editing in disease and development. Such widespread editing variations necessitate an improved understanding of the regulatory mechanisms of RNA editing. Here, we study the roles of >200 RNA-binding proteins (RBPs) in mediating RNA editing in two human cell lines. Using RNA-sequencing and global protein-RNA binding data, we identify a number of RBPs as key regulators of A-to-I editing. These RBPs, such as TDP-43, DROSHA, NF45/90 and Ro60, mediate editing through various mechanisms including regulation of *ADAR1* expression, interaction with ADAR1, and binding to Alu elements. We highlight that editing regulation by Ro60 is consistent with the global up-regulation of RNA editing in systemic lupus erythematosus. Additionally, most key editing regulators act in a cell type-specific manner. Together, our work provides insights for the regulatory mechanisms of RNA editing.

## Abstract (web summary)

Giovanni Quinones-Valdez et al. examined the role of over 200 RNA-binding proteins in mediating A-to-I RNA editing. They identified several RNA-binding proteins that regulate ADAR1 expression, interaction, or binding with Alu elements in a cell type-specific manner.

## Introduction

RNA editing refers to the alteration of RNA sequences through insertion, deletion or substitution of nucleotides^1,2^. In human cells, the most prevalent type of RNA editing is adenosine to inosine editing (A-to-I editing), catalyzed by the protein family adenosine deaminases acting on RNA (ADARs)^3^. A-to-I editing can have profound impacts on gene expression through a wide spectrum of mechanisms, including changing protein-coding sequences^3^, modifying splice sites^4,5^, affecting RNA nuclear export^6^ and altering microRNA sequences or their target sites^3^.

Facilitated by high-throughput RNA sequencing (RNA-seq) methods, millions of A-to-I editing sites have been identified in human cells^7,8^. Although the function of most of these sites remains unknown, the involvement of RNA editing in various biological processes is increasingly appreciated^9^. Recent studies showed that the editing levels of numerous RNA editing sites vary significantly across tissues, developmental stages and disease status^10–12^. These findings prompted many outstanding questions, one of which relates to the regulatory mechanisms that underlie the widespread editing variations. ADAR proteins are the best-known regulators of the human editomes^3^. However, variations in the expression levels of the *ADAR* genes alone can only account for some of the observed editing variations^12–14^. Thus, there is a critical need for identifying and understanding additional regulatory mechanisms of RNA editing.

RNA-binding proteins (RBPs) are important regulators for all steps of RNA maturation. Post-transcriptional RNA processing, such as splicing and polyadenylation, is controlled by the formation of different ribonucleoprotein complexes with RBPs at their core^15,16^. A number of RBPs have been reported to affect RNA editing^14^. For example, some RBPs, such as SRSF9 and RPS14, interact with ADAR2 and affect A-to-I editing of a number of substrates^17^. The protein FMRP (encoded by the FMR1 gene) was shown to affect RNA editing by interacting with the ADAR proteins in multiple organisms^18–21^. Another ADAR1-interacting protein, DICER, was reported to inhibit ADAR1 editing activity in vitro^22^. In addition to ADAR-interacting proteins, other RBPs may affect editing by influencing the double-stranded RNA (dsRNA) substrates of ADAR or the interaction between ADAR and dsRNAs. For example, several RNA helicases, including RNA helicase A (DHX9) and DDX15, were reported to repress RNA editing, presumably by disrupting dsRNA structures^17,23^. The catalytically inactive ADAR protein, ADAR3, represses RNA editing by competitively binding to dsRNA targets in both human cells and *C*. *elegans*^24,25^. Another dsRNA-binding protein, Staufen, binds to numerous inverted Alu repeats^26^, the most prevalent type of ADAR1 substrates in human cells. As a result, Staufen may also regulate RNA editing, although this topic needs further investigation.

It was estimated that more than 3000 RBPs exist in human cells^27^. Although the function of the majority of RBPs is unknown, it is now clear that many RBPs play a role in multiple steps of post-transcriptional RNA processing^15^. However, compared to other processes such as splicing, for which a large number of splicing factors have been cataloged, the number of proteins known to regulate RNA editing remains relatively small. Nevertheless, it is known that ADAR1 potentially interacts with numerous other proteins^28^ and many RBPs may recognize Alu elements or dsRNA targets^14,29,30^. Thus, it is very likely that additional editing regulators remain to be uncovered, which will help to explain the observed editome variability in diseases, between cell types and developmental stages. To this end, we carried out a systematic analysis of the potential involvement of a large panel of RBPs in regulating RNA editing in human cells. We report a number of RBPs as key regulators of RNA editing, most of which function in a cell type-specific manner. Our findings greatly expand the repertoire of known RBPs as RNA editing regulators.

## Results

### Global analysis of RNA editing upon knockdown of >200 RBPs

To examine potential regulatory mechanisms of RNA editing, we analyzed a large number of RNA-seq datasets generated upon knockdown of hundreds of RBPs as part of the ENCODE project^31^. Specifically, data from two human cell lines, K562 (chronic myelogenous leukemia) and HepG2 (liver hepatocellular carcinoma) were included. Individual knockdown experiments were carried out for 222 and 225 RBPs in K562 and HepG2 cells, respectively. For each RBP, two biological replicates of knockdown were carried out, followed by polyA-selected RNA-seq, which were accompanied by two replicates of control experiments. An average of 33.5 million pairs of reads (2 × 100 or 2 × 101 nt) were obtained for each knockdown or control replicate. To identify RNA editing sites, the RNA-seq data were analyzed using our previously developed methods^32–35^, followed by batch-normalization (see Methods).

A total of 893,701 and 444,263 distinct editing sites were identified in the K562 and HepG2 samples, respectively. In a single dataset, the number of predicted editing sites ranged from 226 to 16,657, which approximately correlated with RNA-seq read coverage of the samples (Supplementary Fig. 1a). An average of 92% of each sample’s editing sites sample were of the A-to-G type, consistent with A-to-I editing (Supplementary Fig. 1a). This high percentage suggests a high accuracy of our RNA editing identification method, as previously shown^35^. As expected, the majority of editing sites were located in Alu regions (Supplementary Fig. 1b), and introns or 3’ UTRs (Supplementary Fig. 1c). Among all distinct A-to-G editing sites from the two cell lines, 63 and 69% overlapped those in the RADAR^8^ and REDIPortal^7^ databases, respectively. In this study, we restricted all subsequent analyses to A-to-G sites in order to focus on ADAR-catalyzed editing.

### The landscape of differential editing upon RBP knockdown

**Fig. 1:**
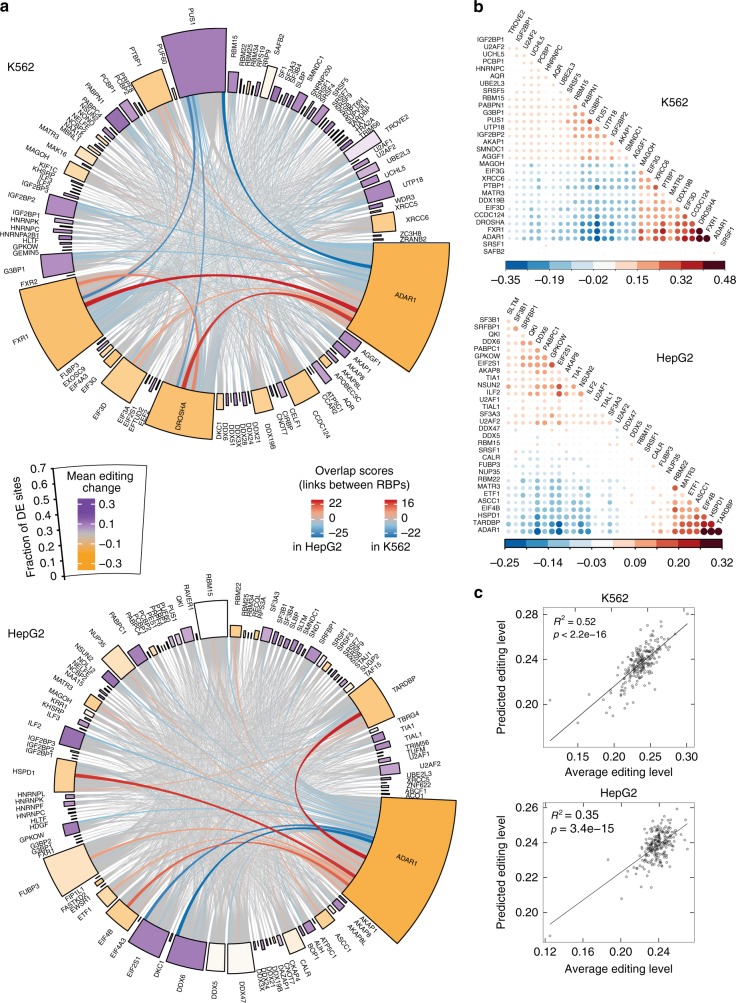
Global overview of RNA editing regulation by RBPs. **a** CIRCOS plot illustrating differential editing patterns upon knockdown of each RBP in each cell line. For each cell line, 100 RBPs are shown as those with the highest percentage of differentially edited sites among all testable sites (represented by the height of the outer box). The color of the box denotes the average changes in editing levels of differentially edited sites relative to controls upon RBP knockdown. For the links between RBPs, the thickness and color of the line both reflect an overlap score, that is, the fraction of shared differentially edited sites among shared testable sites between two RBPs (Methods). Positive overlap scores reflect concordant direction in the editing changes induced by the pair of RBPs, while negative values represent the opposite. The width of the box is set automatically to accommodate all the links associated with each box. ADAR1 has the greatest impact on global editing in both cell lines, which serves as a positive control. **b** Hierarchical clustering of RBPs using pair-wise directional agreement scores (Methods). The top 31 RBPs with the highest percentage of differentially edited sites among all testable sites per cell line are shown. The size of the dot and its color both reflect the directional agreement score. These RBPs cluster in two main groups composed of those associated with positive or negative editing changes upon their knockdown. **c** Correlation between actual average editing levels per sample and predicted RNA editing levels calculated via linear regression of gene expression levels of the top 15 RBPs in each cell line (including ADAR1). Each dot represents one sample and all RBP knockdown samples are included. *R*^2^ and *p* values were calculated by Pearson correlation. The expression of these RBPs explained about 35 and 52% of the total variance in editing in HepG2 and K562 cells, respectively

Next, to assess the impact of RBPs on RNA editing, we identified differentially edited sites upon knockdown of each of these RBPs in each cell line (see Methods). We observed that different RBPs induced variable degrees of editing changes, ranging from being negligible to affecting nearly 50% of all testable sites (see Methods) (Fig. 1a, Supplementary Fig. 2). As a positive control, ADAR1 knockdown induced the most widespread editing reduction among all RBPs, supporting the effectiveness of our methods.

A comparison of differentially edited sites associated with different RBPs revealed that ADAR1 shared many sites with other RBPs in both cell lines (Fig. 1a, links between RBPs), consistent with the fact that ADAR1 is a main catalytic enzyme of A-to-I editing. It is also apparent that a small number of RBPs were associated with relatively high levels of editing changes in either positive or negative direction (Fig. 1a, Supplementary Fig. 2c, f). Specifically, 31 and 15 RBPs in K562 and HepG2 cells, respectively, had at least 10% of all testable sites as differentially edited sites (Supplementary Fig. 2a, d). Examples of such RBPs include ADAR1, FXR1, DROSHA and TARDBP. Notably, some of these RBPs shared many differentially edited sites (Fig. 1a, links between RBPs, and Supplementary Fig. 3). For the union of differentially edited sites of a pair of RBPs, we calculated a directional agreement score to evaluate the concordance of the directions of editing changes associated with two RBPs (Methods). Two small clusters of RBPs showed relatively high (same directional changes) or low (opposite directional changes) agreement scores with ADAR1, supporting possible existence of both enhancers and repressors of editing (Fig. 1b). Using a linear regression model, we estimated that the top 15 RBPs (including ADAR1), each of which affected ≥10% of editing sites in K562, together accounted for 52% of editing variation in this cell line, and 35% in HepG2 (Fig. 1c). This percentage remains high even if the most influential samples (such as ADAR1 and DROSHA knockdown) were excluded (Supplementary Fig. 4a). Using ADAR1 expression alone in the regression accounted for 6% and 15% of editing variation in K562 and HepG2, respectively (Supplementary Fig. 4b). In contrast, this percentage is 43.8 and 8.7% if 14 RBPs (except ADAR1) were used in K562 and HepG2, respectively (Supplementary Fig. 4c). Moreover, the inclusion of an interaction term between ADAR1 and the other RBPs increased this percentage by about 10% in each cell line, although this interaction term was not statistically significant (Supplementary Fig. 4d). Together, these results support that RNA editing is regulated by auxiliary proteins besides ADAR1 and the functional impact of such proteins is likely cell type-specific.

To more systematically examine the correlative relationship among RBPs, we calculated the correlation of editing changes of the differentially edited sites associated with a pair of RBPs, respectively. For this analysis, we included RBPs whose knockdown induced differential editing among ≥2% of all testable sites. We then applied hierarchical clustering on the correlation coefficients (Supplementary Fig. 5a, b). In each cell line, we observed one small cluster mainly containing RBPs (including ADAR1) associated with the greatest reduction in editing levels upon their knockdown. A similar pattern was observed when clustering RBPs with WGCNA (weighted-gene co-expression networks) (Supplementary Fig. 6a, b), a more robust statistical framework than hierarchical clustering^36^. Compared to RBPs that induced editing reduction upon knockdown, those associated with editing up-regulation upon their knockdown did not cluster strongly. Importantly, experimental batches did not confound the clusters in either hierarchical clustering or WGCNA (Supplementary Fig. 5, Supplementary Fig. 6). Together, these results suggest that a relatively small number of RBPs are associated with observable editing changes that are correlated with each other.

Since many RBPs may contribute to multiple RNA processing mechanisms^15^, the RNA editing changes observed upon knockdown of an RBP may not reflect a direct involvement of the RBP in regulating RNA editing. To further understand the underlying processes, we next examined whether an RBP may impose editing changes through three types of possible mechanisms: by regulating ADAR1 expression; by interacting with the ADAR proteins; or by binding to similar RNA substrates as the ADAR proteins.

### TARDBP as a regulator of ADAR1 expression

**Fig. 2:**
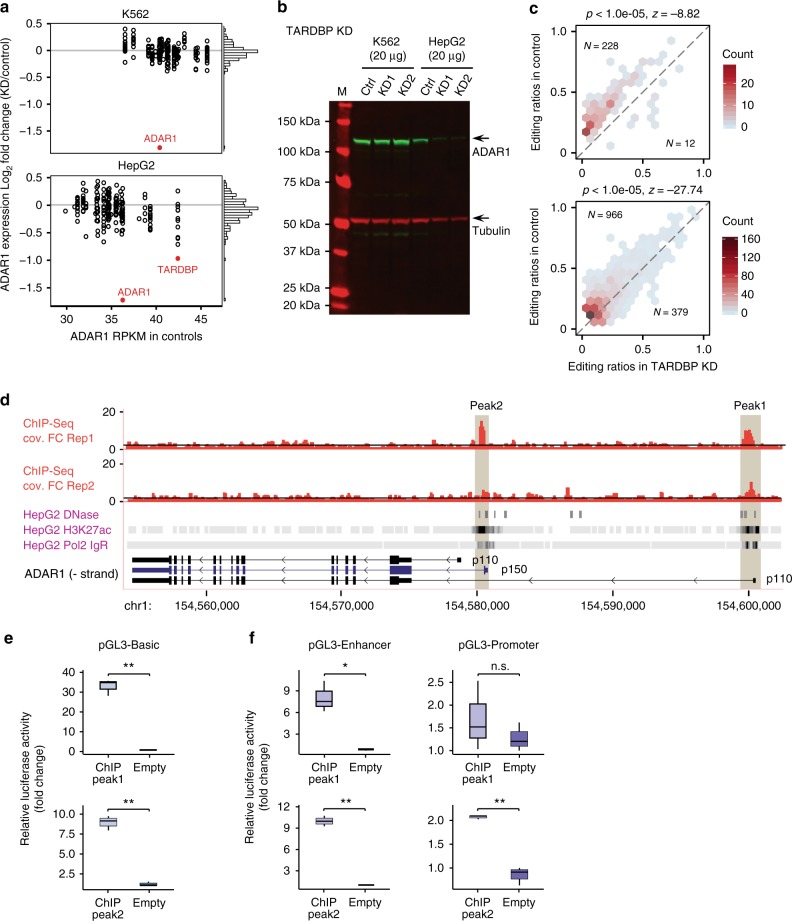
Regulation of ADAR1 expression by TARDBP. **a** Differential ADAR1 mRNA expression upon RBP knockdown in K562 and HepG2 cells compared to controls. Histograms on the right show the distributions of log-fold-change (LFC) values. TARDBP is the only RBP whose knockdown caused differential expression of ADAR1 (absolute value of LFC knockdown/Control ≥1 and DESeq *q*-value < 10^−9^). **b** Western blot of shRNA-mediated TARDBP knockdown and control (Ctrl) cells. Blots were probed with antibodies detecting ADAR1 and Tubulin (as a control). TARDBP knockdown significantly reduced ADAR1 expression in HepG2 cells only. **c** Editing ratios in TARDBP knockdown samples in HepG2 compared to their respective control values. The numbers (*N*) of editing sites with decreased and increased editing upon TARDBP knockdown are shown. The top panel includes only differentially edited sites and the bottom panel shows all testable editing sites. *P*-values and *z* scores were calculated using a bootstrap sampling strategy to evaluate the bias in the numbers of up- vs. down-regulated editing sites (Methods). Knockdown of TARDBP caused a global downregulation in editing levels. **d** TDP-43 (encoded by the TARDBP gene) ChIP-seq peaks in HepG2 cells overlapping ADAR1 transcripts. Fold change of read coverage relative to input control is shown for two replicated experiments (Rep1 and 2). Black line denotes fold change = 2. Three representative ADAR1 transcripts (RefSeq annotation) are shown, coding for the p110 and p150 forms of the ADAR1 protein. In addition, H3K27Ac, DNase hypersensitivity data in HepG2 cells and RNA polymerase 2 binding data generated by the ENCODE consortium are shown. **e**, **f** Luciferase assays of a series of pGL3 constructs containing the TDP-43 ChIP peak regions. The ChIP sequence for peak1 and peak2 were built into the construct. Basic pGL3 construct (**e**) and pGL3-Enhancer (lacking the SV40 promoter) and pGL3-Promoter (lacking the SV40 enhancer) constructs (**f**) are shown. The ratio of firefly luciferase to renilla luciferase was calculated for each experiment. The mean value (3 replicates) for each test construct was normalized to the activity of the empty vector. (Unpaired, two-tailed Student’s *t*-test, *P < 0.05, **P < 0.01, n.s.: not significant)

To examine whether any RBPs may regulate ADAR expression, we analyzed ADAR1/2/3 mRNA expression levels in different RBP knockdown samples (Fig. 2a, Supplementary Fig. 7a, b). ADAR1 is much more abundant than ADAR2 and ADAR3 in both cell lines. Thus, we next focused on potential regulators of ADAR1. We observed that most RBPs did not cause significant changes in ADAR1 mRNA expression. One exception is the gene TARDBP, whose knockdown induced about two-fold reduction in ADAR1 mRNA level in HepG2 cells. In addition, Western blot analysis confirmed that ADAR1 protein level was also reduced upon TARDBP knockdown in HepG2 cells, but not in K562 cells (Fig. 2b). Consistent with the observed ADAR1 expression changes, TARDBP knockdown induced a global reduction in RNA editing levels in HepG2 cells (Fig. 2c), but not in K562 cells (Fig. 1a).

TARDBP encodes for the TDP-43 protein that binds to both DNA and RNA sequences^37^. TDP-43 is known to regulate transcription and multiple RNA processing steps^37^. To examine the potential regulatory mechanisms of TDP-43 on ADAR1 expression, we asked whether it may regulate ADAR1 transcription. We analyzed existing chromatin immunoprecipitation (ChIP-Seq) data of TDP-43 (HepG2 cells) from the ENCODE consortium^31^ (Fig. 2d, Supplementary Fig. 7c). A significant ChIP peak (FDR = 0.001, peak 1) was observed overlapping the first exon of a p110 isoform of ADAR1. A second peak (FDR = 0.002, peak 2), although only significant in one replicate, was found upstream of the first exon of another p110 isoform. Note that the latter peak also overlaps the first exon of the p150 isoform of ADAR1. However, ADAR1 p150 expression is undetectable in HepG2 (Fig. 2b). Both ChIP peaks are located in open chromatin regions as denoted by the presence of H3K27Ac marks, DNase I hypersensitive sites and transcription factors binding clusters (Pol2 IgR) (Fig. 2d).

Based on these data, we hypothesized that the ChIP peak regions are regulatory elements that control transcription of ADAR1. To test this hypothesis, we cloned these regions, respectively, into different luciferase reporters to test whether they may serve as promoter or enhancer elements. These constructs were transiently transfected into HepG2 cells, followed by measurement of luciferase activity. Using the PGL3-Basic vector, we observed a significant increase in luciferase activity with the inclusion of either peak region, compared to empty vectors (Fig. 2e). Furthermore, in the PGL3-Enhancer vector that lacks the SV40 promoter, both peak regions induced higher luciferase activity than controls (Fig. 2f). In contrast, only the second peak region induced significant luciferase activity in the PGL3-Promoter vector that lacks the SV40 enhancer (Fig. 2f). We additionally validated the binding of TDP-43 to the reporter construct using ChIP followed by real-time and semi-quantitative PCR (Supplementary Fig. 7d, e and f). The PCR amplification of the TDP-43 Immunoprecipitation products showed significant enrichment of the TDP-43 binding sequences used in the reporter assay (Supplementary Fig. 7e and f). Together, these results suggest that TDP-43 binds to multiple regulatory regions of the ADAR1 gene that may serve as promoters or enhancers.

### ADAR1-interacting RBPs as RNA editing regulators

ADAR1 is known to interact with a large number of proteins^28^ (Supplementary Table 1). A prevailing question in the field is whether ADAR1’s interacting partners may confer regulation on RNA editing. To examine this question, we started from known ADAR1-interacting proteins, and asked: whether knockdown of a protein induced a considerable amount of RNA editing changes; and whether this protein binds significantly close to the differential RNA editing sites observed upon its knockdown. The confirmation of the second question serves as a strong indication of the direct involvement of a protein in modulating RNA editing. Such proteins likely affect RNA editing through their known interacting relationships with ADAR1, although the exact mechanisms need to be investigated in the future.

**Fig. 3:**
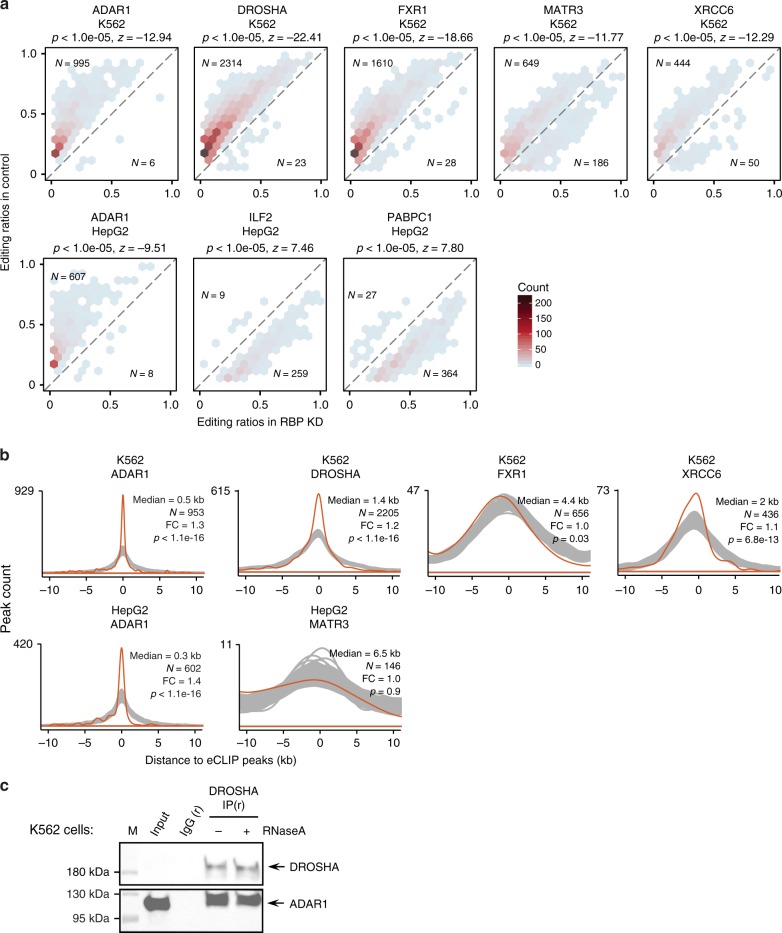
Regulation of RNA editing by ADAR1-interacting RBPs. **a** Editing ratios of differentially edited sites in RBP knockdown samples and their corresponding controls for ADAR1 and ADAR1-interacting proteins, similar as Fig. 2c. **b** Distribution of distances between eCLIP peaks and their closest differentially edited sites (orange) or control sites (gray) (Methods). The median distance is shown. N represents the number of differentially edited sites used in the calculation. Calculations of fold change and *p* values are described in the Methods section. ADAR1, DROSHA and XRCC6 bind significantly closer to differentially edited sites than to control sites. **c** Co-IP experiment with and without RNase A treatment in K562 cells demonstrates interaction between ADAR1 and DROSHA. Immunoprecipitation was performed using DROSHA antibody or corresponding rabbit (r) isotype IgG. Blots for immunoprecipitation samples were probed with antibodies detecting ADAR1 and DROSHA

Our study included 18 known ADAR1-interacting proteins whose expression was knockdown in at least one cell line. The majority of known ADAR1-interacting proteins induced differential editing in <10% of the associated testable editing sites upon their knockdown in K562 or HepG2 cells (Supplementary Fig. 8a). This observation suggests that not all ADAR-interacting partners influence RNA editing extensively. Nevertheless, a small number of RBPs were associated with considerable editing changes (≥10% of all testable sites) upon their respective knockdown, including DROSHA, FXR1, XRCC6 and MATR3 in K562 cells, ILF2 and PABPC1 in HepG2 cells (Fig. 3a).

Next, we asked whether the above proteins might be direct regulators of RNA editing by examining the RBP binding locations relative to differentially edited sites. Although not a necessary requirement for direct regulators of RNA editing, a significantly close distance between RBP binding and differentially edited sites provides strong support for a direct regulatory relationship. To examine the global protein-RNA binding patterns, we analyzed the enhanced crosslinking immunoprecipitation (eCLIP) data for the above proteins generated by the ENCODE project^31^ (except ILF2 and PABPC1 for which eCLIP data are not available) (Fig. 3b). As a positive control, we included our previously published ADAR1 CLIP-Seq data from the U87MG cells^38^ in this analysis. Although ADAR1 CLIP was generated using a different cell type, the CLIP peaks were significantly closer than expected by chance to differentially edited sites observed in K562 or HepG2 cells upon ADAR1 knockdown. This data supports the validity of this analysis.

Among all proteins included in this analysis, DROSHA demonstrated the most significant relationship between protein binding and protein knockdown-induced differential editing (Fig. 3a, b). DROSHA is best known to bind to double-stranded primary microRNA (miRNA) structures and mediate miRNA biogenesis in the nucleus^39^. In this study, we observed that DROSHA is one of the RBPs that induced the strongest reduction in RNA editing upon its knockdown in K562 cells, affecting ~30% of all testable editing sites (Figs 1a and 3a). To further confirm the involvement of DROSHA in regulating RNA editing, we carried out an immunoprecipitation experiment for this protein, followed by ADAR1 immunoblotting in K562 cells (Fig. 3c, Supplementary Fig. 8b). Note that DROSHA’s endogenous expression in K562 cells is relatively low, which precluded its detection in the input sample. Nonetheless, the expression of DROSHA is clearly detectable in the IP samples. The presence of ADAR1 in DROSHA IP samples support that these two proteins interact with each other in K562 cells. In addition, RNase A treatment did not affect the observed interaction, suggesting that the interaction between ADAR1 and DROSHA does not depend on single-stranded RNA (the main target of RNase A). This result is consistent with our previous observation in HeLa cells^38^. Lastly, we confirmed that DROSHA knockdown did not induce observable change in ADAR1 protein expression in K562 cells (Supplementary Fig. 8c). Together, our data support that DROSHA is a strong enhancer of RNA editing, most likely by interacting with ADAR1 in the nucleus.

In addition to DROSHA, ILF2 and ILF3 (also called NF45 and NF90) are likely direct regulators of RNA editing via ADAR1 interactions. Both proteins are well-known RNA-dependent interacting proteins of ADAR1^40,41^. These proteins are localized in the nucleus^42^ and form complexes to regulate multiple aspects of RNA metabolism^40,43,44^. We observed that ILF2 knockdown in HepG2 cells caused up-regulation of editing levels in 13% of testable sites (Fig. 3a, Supplementary Fig. 8a). Its impact on editing in K562 cells is less pronounced than in HepG2 cells, but the same predominant direction of up-regulation was observed, which affected 5% of testable sites (Supplementary Fig. 8a). Similarly, ILF3 knockdown induced up-regulation of editing levels in 4% of sites in both cell lines (Supplementary Fig. 8a). ILF3 (but not ILF2) eCLIP-seq data are available in both K562 and HepG2 cells. We observed that ILF3 binds significantly closer to differentially edited sites than expected by chance in both cell lines (Supplementary Fig. 8d). Together with previous findings that ILF2 and ILF3 form protein complexes and interact with ADAR1^40,41^, our data support a model where these proteins repress RNA editing by interacting with ADAR1 and binding close to ADAR1’s target sequences. In addition, compared to IFL3, ILF2 may play a more direct role in influencing the editing outcomes of ADAR1.

### Alu-binding RBPs as RNA editing regulators

**Fig. 4:**
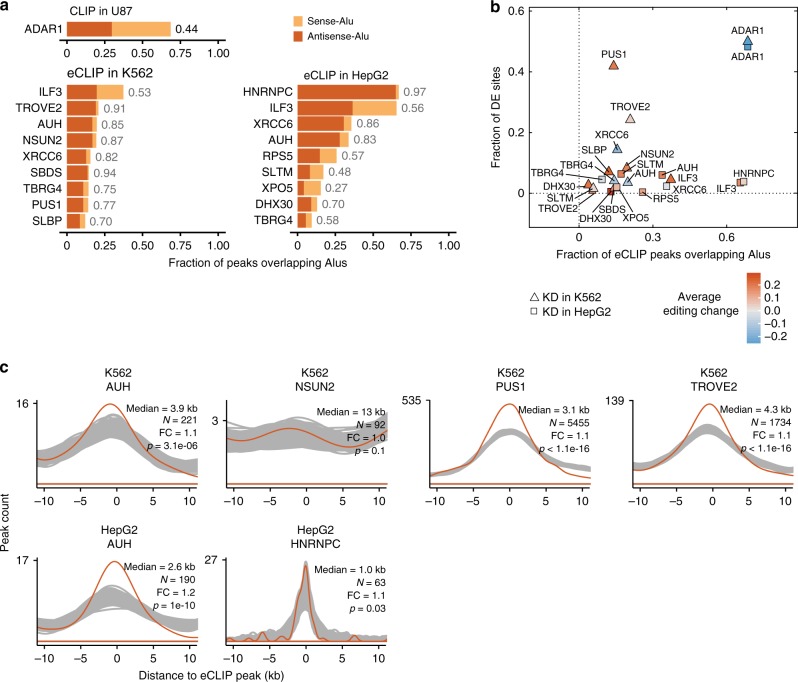
Regulation of RNA editing by Alu-binding RBPs. **a** Top RBPs ranked by their fractions of eCLIP peaks that overlap Alu elements. RBPs were required to have both RNA-seq and eCLIP-seq data in the same cell line. ADAR CLIP-seq data was obtained in the U87MG cells. Sense and antisense Alus denote Alu elements with consensus sequence on the same or opposite strand as the CLIP or eCLIP peaks, respectively. The fraction of antisense Alus among all Alu-overlapping peaks is shown for each protein (next to the bar). **b** For the RBPs in **a**, comparison between the fraction of differentially edited sites among all testable sites and the fraction of eCLIP peaks that overlap Alu elements. The dots are colored according to the average editing changes of differentially edited sites (knockdown-control). There is no direct correlation between Alu-binding frequency and editing regulation for the tested RBPs. **c** Distribution of distances between eCLIP peaks and their closest differentially edited sites (orange) or control sites (gray) (Methods), for RBPs in **a** with eCLIP data, calculated similarly as in Fig. 3b. AUH, PUS1 and TROVE2 bind significantly closer to differentially edited sites than to control sites

Alu sequences, especially inverted Alu pairs, form dsRNA structures, which are the main ADAR1 substrates for RNA editing in human cells^3,9^. One natural question is whether Alu-binding RBPs in general may regulate RNA editing by facilitating or inhibiting the interaction between ADAR1 and its dsRNA substrates. To address this question, we first analyzed all available ENCODE eCLIP-seq datasets to identify Alu-binding RBPs (Methods). We observed that a small number of RBPs are associated with high levels of Alu-binding, manifested as the high percentage of eCLIP peaks overlapping sense or antisense Alu elements (Fig. 4a). As expected, ADAR1 showed the highest level of Alu-binding among all proteins, despite the fact that ADAR1 CLIP was generated using a different cell line (U87MG cells)^38^. Notably, some proteins (such as hnRNP C) demonstrated a substantial bias for preference to either sense or antisense Alus, compared to the background sense/antisense Alu composition in expressed genes (~44% sense, ~56% antisense Alus in HepG2 and K562). In contrast, the sense/antisense Alu compositions of ILF3 and ADAR1 peaks are similar to the background, which may indicate that their binding specificity relies on RNA structures more than on the specific sequences.

Next, we asked whether the extent of Alu-binding correlates with the level of impact of each RBP on RNA editing. Surprisingly, there is little correlation between these two variables (Fig. 4b). In addition, the direction of editing changes upon RBP knockdown did not show consistent trend for these proteins (Supplementary Fig. 9). These observations suggest that Alu-binding alone is not sufficient for an RBP to influence ADAR1 editing. For a subset of these RBPs, eCLIP-seq data are available (Fig. 4c). We observed that the binding sites of ILF3 (Supplementary Fig. 8d), XRCC6 (Fig. 3b), TROVE2, AUH and PUS1 are significantly closer to differentially edited sites than expected by chance (Fig. 4c). Note that ILF3 and XRCC6 are also known ADAR1-interacting proteins (Supplementary Table 1), as described in the section above. The third gene, TROVE2, affects ~25% of all testable editing sites, with a bias toward up-regulated editing levels upon TROVE2 knockdown (Fig. 4b). We therefore further investigated the possible involvement of this protein in RNA editing regulation, as presented in the next section.

### Alu-binding protein Ro60 (TROVE2) in RNA editing regulation

**Fig. 5:**
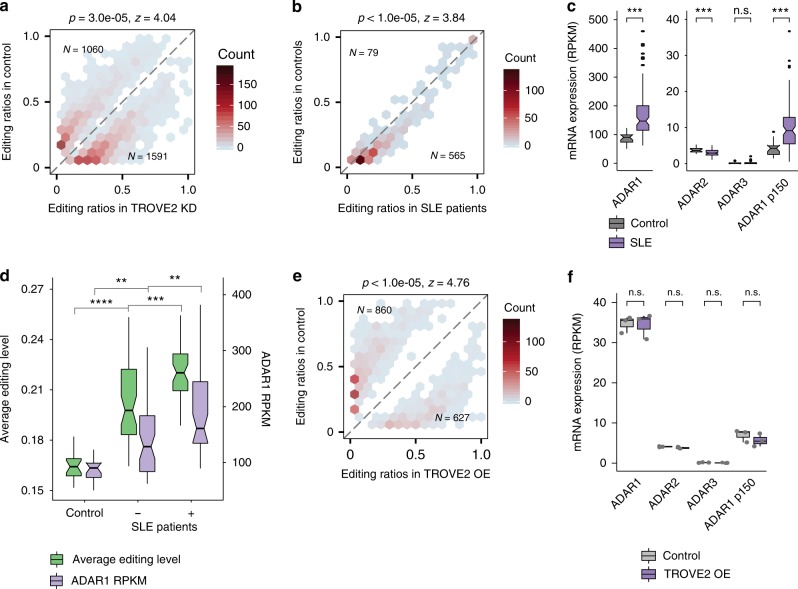
Regulation of RNA editing by TROVE2. **a** Editing ratios of differentially edited sites in TROVE2 knockdown samples and the corresponding controls in K562 cells, similar as Fig. 2c. **b** Similar as **a**, editing ratios of differentially edited sites in blood samples of SLE patients and control subjects. The data demonstrate reduced editing levels in SLE patients. **c** mRNA expression of ADAR1, ADAR2, ADAR3 and the ADAR1 p150 isoform in blood samples of SLE patients and control subjects (18 Controls, 99 SLE Patients, *p* values were calculated using two-tailed Wilcoxon ranksum test, ***P < 0.001, ****P < 10^−5^). **d** Average editing levels and ADAR1 mRNA expression (RPKM) of control subjects, SLE patients with medium to high Ro60 antibody levels (+) and SLE patients without detectable Ro60 antibody levels (−). Increasing levels of Ro60 antibody correlated with higher ADAR1 expression and editing levels (*P*-values were calculated using two-tailed a Wilcoxon rank sum test and corrected for multiple testing using Bonferroni correction, **P < 0.01, ***P < 0.001, ****P < 10^−8^). **e** Similar as **a**, editing levels of differentially edited sites in TROVE2 overexpression samples and the corresponding controls in K562 cells. Overexpression of TROVE2 led to a bias toward lower editing levels. **f** mRNA expression of ADAR genes in TROVE2 overexpression samples in K562 cells (*N* = 3 biological replicates, *P* values were calculated using a two-tailed Wilcoxon ranksum test). No significant change was observed in ADAR expression in TROVE2 overexpression samples compared to controls

TROVE2 encodes for the protein Ro60, which is present in both the nucleus and cytoplasm of vertebrate cells^45^. Anti-Ro60 antibodies occur in many patients with systemic lupus erythematosus (SLE), an autoimmune disease characterized by interferon activation, autoantibodies and multi-organ tissue destruction^46^. We analyzed RNA editing patterns using RNA-seq data derived from the blood samples of 99 SLE patients and 18 controls^47^. Consistent with our findings in K562 cells (Fig. 5a), SLE samples, many with loss of Ro60 function, showed a predominant bias of upregulated RNA editing levels (Fig. 5b), which was also reported in a recent study^48^. Moreover, consistent with interferon activation in SLE, we observed that ADAR1, particularly the interferon-inducible p150 isoform, was significantly overexpressed in SLE patients (Fig. 5c).

Based on the data above, the up-regulation of RNA editing in SLE may be due to one or both of the following mechanisms: up-regulated ADAR1 expression as a result of interferon response; or loss of Ro60, a repressor of RNA editing via Alu-binding. We observed a correlated pattern between the loss of Ro60 and up-regulation of ADAR1, both correlating with up-regulated RNA editing levels (Fig. 5d). To distinguish these two models, we obtained RNA-seq data from K562 cells following TROVE2 overexpression. Compared to control cells, TROVE2-overexpressing cells showed a significant bias toward reduced editing levels (Fig. 5e), while no significant change in ADAR expression was observed (Fig. 5f). It should be noted that ADAR expression levels did not change upon TROVE2 knockdown in K562 cells either (Supplementary Fig. 10). Therefore, our data support a direct role of TROVE2 in repressing RNA editing, most likely by its interaction with Alu elements. Notably, the observed up-regulation of RNA editing in SLE patients likely reflects contribution by both loss of Ro60 function and ADAR1 up-regulation. As a result, the extent of RNA editing changes in SLE is much more pronounced than those observed in TROVE2 knockdown or overexpressing cells (Fig. 5a, b, e), consistent with the lack of ADAR1 expression changes in the latter groups.

### Cell type differences in RNA editing regulation

While examining RBPs in the above categories, we observed that the two cell lines, K562 and HepG2, were often associated with different RBPs that imposed the largest impact on editing. In total, 199 RBPs have RNA-seq data generated from both cell lines. However, only 3 RBPs, including ADAR1, were found to affect editing in ≥10% of all testable sites in both cell lines. Thus, we next examined whether the impact of RBPs on RNA editing is different between these two cell lines. For this analysis, we focused on the 35 RBPs with available data in both cell lines whose knockdown induced differential editing changes in ≥10% of the testable sites in at least one cell line (Supplementary Fig. 11a). It should be noted that, for the majority of these RBPs, their possible mechanisms of action on editing are not clear, including whether the observed editing changes are direct or indirect effects of RBP knockdown.

**Fig. 6:**
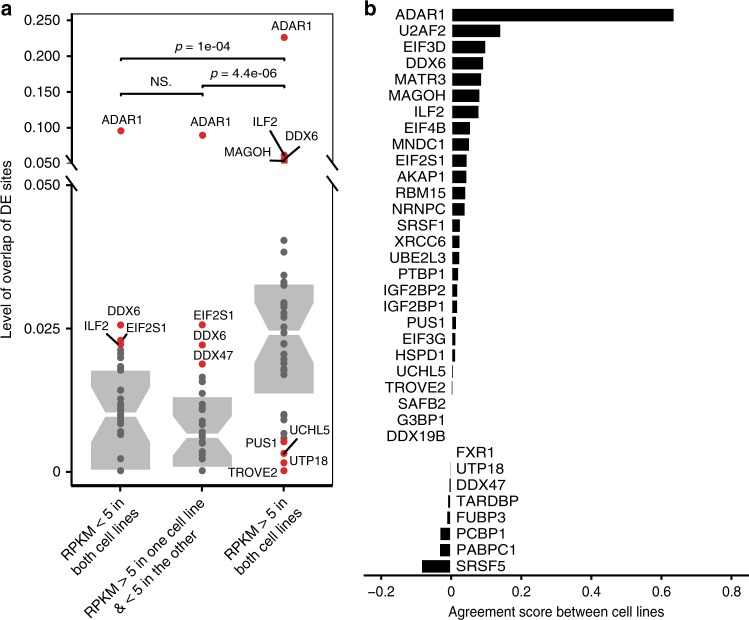
Cell type-specific impact of RBPs on RNA editing. **a** Overlap of differentially edited sites between K562 and HepG2 cells for RBPs with at least 10% testable sites being differentially edited sites in at least one cell line. The differentially edited sites are separated into 3 groups based on the expression levels (RPKM) of the corresponding genes in the two cell lines. In each group, a ratio (level of overlap) was calculated for each RBP between the number of shared differentially edited sites and the number of the union of differentially edited sites between the two cell lines. *P* values were calculated using Wilcoxon rank sum test. Editing sites in highly expressed genes common to the two cell lines had the highest overlap between the two cell lines among the 3 groups of genes. **b** For RBPs in **a**, directional agreement scores (Methods) of their differentially edited sites between K562 and HepG2 cells

Since RNA editing is only observable in expressed RNA, one main factor underlying cell type-specificity in editing is the availability of the target transcripts. Thus, we first examined the between-cell-line overlap of differentially edited sites associated with each RBP in groups of genes stratified by their expression levels. As expected, the overlap of differentially edited sites is much higher in genes relatively highly expressed in both cell lines than those that are high in only one cell line (Fig. 6a). Thus, cell type-specific gene expression contributes to the observed differences in editing profiles between the two cell lines.

To further compare RBP knockdown-induced editing changes between the two cell lines, we identified the set of testable editing sites (total read coverage ≥5 per replicate and editing ratio ≥10% in either knockdown or control) common to both cell lines for each of the 35 RBPs. Indeed, common testable editing sites for most RBPs only constitute <50% of all testable sites in each cell line (Supplementary Fig. 11b), again reflecting differences in gene expression levels. Among these common sites, the fraction of differentially edited sites for each RBP is comparable to that among all testable sites in each cell line (Supplementary Fig. 11c vs. Supplementary Fig. 11a). Next, we evaluated the concordance of editing changes in the common sites between the two cell lines. To this end, we calculated the directional agreement scores as defined in Fig. 1b (Methods). A small number of RBPs, such as SRSF5, PABPC1, and PCBP1, had apparent opposite directions (positive or negative) in editing changes upon their knockdown in the two cell lines (Supplementary Fig. 11c), which resulted in negative agreement scores (Fig. 6b). Additionally, the majority of these RBPs had low agreement scores (e.g., 25 RBP with absolute score <0.05, meaning that less than 5% of their differentially edited sites agree), including TROVE2 and TARDBP. Together, our analyses of common testable sites suggest that the (direct or indirect) impact of RBPs on RNA editing is different depending on the cell type.

## Discussion

We report a global study to identify RBPs as regulators of A-to-I editing in human cells. Using hundreds of RNA-seq datasets derived upon knockdown of individual RBPs, we investigated the influence of each RBP on RNA editing in K562 and HepG2 cells. Complemented by protein-RNA binding analyses using eCLIP-seq data and experimental validations, our study yielded a number of findings that help to fill in the significant gap in our understanding of additional regulators of RNA editing beyond the ADAR proteins.

An important observation of this study is that, among >200 RBPs analyzed in each cell line, only a small number of proteins caused substantial changes in RNA editing upon their knockdown. Since most RBPs contribute to multiple aspects of RNA processing and regulation^15,16^, it is not surprising that loss of an RBP may cause a myriad of changes in gene expression, including RNA editing, directly or indirectly. Indeed, our data showed that for the vast majority of RBPs, there always existed a small fraction of editing sites with altered editing levels upon RBP knockdown. Such small degrees of changes are most likely consequences of alterations in other aspects of RNA regulation that sporadically and indirectly correlated with an observed RNA editing change. For example, changes in alternative splicing caused by RBP knockdown may affect the observed level of editing for certain sites in the intron. Therefore, we reason that direct regulators of RNA editing, those that affect the expression, function or protein-RNA interactions of ADAR proteins, should cause considerable editing changes that are relatively widespread. It should be noted that the reverse may not always hold—some proteins associated with large editing changes may not be direct regulators of RNA editing.

We focused on three categories of potential direct regulators of RNA editing: proteins that regulate ADAR expression, interact with ADAR1, or bind to Alu elements. One immediate observation is that not all ADAR-interacting or Alu-binding proteins influence RNA editing significantly. Based on previous studies, ADAR1 interacts with many RBPs^28^. However, ADAR-interaction studies were carried out in specific cell types. It is possible that these protein–protein interactions are highly cell type-specific, which may explain the lack of RNA editing changes upon knockdown of many known ADAR-interacting proteins in K562 or HepG2. In addition, ADAR proteins were shown to affect post-transcriptional processes other than RNA editing^38^. Thus, another explanation for our observation is that some ADAR-interacting proteins may affect other aspects of ADAR1 function. Similarly, Alu-binding proteins may not affect RNA editing if their interactions with Alus are independent of ADAR1 or if they affect other aspects of ADAR1 function.

Within the above categories, we highlighted a few RBPs with significant impact on RNA editing, including DROSHA, ILF2/3, TARDBP and TROVE2. In our previous study^38^, we reported that ADAR1 interacts with DROSHA and enhances miRNA production in HeLa cells. Here, we confirmed the interaction between DROSHA and ADAR1 in K562 cells (Fig. 3c). This interaction is consistent with the observed significant reduction in RNA editing upon DROSHA knockdown in K562 cells (Fig. 3a) and the closer distance than expected by chance between DROSHA binding and the differentially edited sites (Fig. 3b). Together, these results suggest that the interaction between DROSHA and ADAR1 enhances the primary functions of these proteins reciprocally. Interestingly, another family of well-known ADAR1-interacting proteins, ILF2 and ILF3, were also reported to affect miRNA biogenesis^44,49^, the knockdown of which caused reduction of RNA editing. Therefore, RNA editing and miRNA biogenesis may be regulated by a common set of RBPs, likely due to the involvement of double-stranded RNA structures in both pathways.

Another protein with a significant role in editing regulation is Ro60 (encoded by the gene TROVE2). We observed that Ro60 binds to Alu elements and TROVE2 knockdown induced an increase in RNA editing for more than 1000 editing sites in K562 cells. This editing change was recapitulated in SLE patients with loss of Ro60 function. The patient samples showed a substantial change in RNA editing, to a higher extent than that observed in K562 cells, possibly due to the combined impact of ADAR1 p150 upregulation and Ro60 loss of function in SLE. Another disease-related protein with involvement in regulating RNA editing is TDP-43 (encoded by the gene TARDBP). TDP-43 is a key player in the pathogenesis of Amyotrophic Lateral Sclerosis (ALS)^50^, a neurodegenerative disease caused by the aggregation of TDP-43 in the cytoplasm of neurons^37^. We observed that TDP-43 enhances ADAR1 transcription, thus influencing the global levels of RNA editing. For both SLE and ALS, further studies are needed to better understand how aberrant RNA editing profiles may contribute to the disease processes.

In addition to the RBPs highlighted above, there are a number of other proteins that were observed with extensive changes in RNA editing upon their knockdown. For example, FXR1 knockdown led to significant reduction of RNA editing in K562 cells (38% of editing sites were downregulated), but not HepG2 cells (Fig. 1). Indeed, our recent study reported that FXR1 reduces RNA editing in the brain and contributes to hypoediting in Autism brains^21^. Thus, the role of FXR1 in RNA editing depends greatly on the cell type, as similarly observed for other proteins in this study (Fig. 6). In addition to direct regulators of RNA editing or ADAR1 expression, knockdown of some RBPs may cause an apparent editing change due to indirect mechanisms. One previously reported mechanism for such indirect effects is editing-dependent stabilization of mRNAs, mediated by the AGO2-miRNA targeting pathway^13^.

Lastly, it should be noted that many other mechanisms may affect RNA editing, which are not studied in this work, including those executed by RNA helicases^17,23^, snoRNAs^51,52^ or proteins that affect ADAR protein modification, degradation or localization^12,53,54^.

## Methods

### Datasets

Fastq files of RNA-seq data generated following RBP knockdown or control shRNA transfection were downloaded from the ENCODE data portal^31^ (encodeproject.org). Data released between October 2014 and January 2017 is included in this study. These data were generated in 24 and 26 batches in the K562 and HepG2 cell lines, respectively.

RNA-seq reads were aligned using RASER v0.52^34^ against the human genome (hg19) and Ensembl transcriptome^55^ (Release 75), with the parameters *m* = 0.05 and *b* = 0.03. Only uniquely mapped reads were retained for further analysis. Duplicated reads (those with identical start and end coordinates) were removed from the alignment files.

### Identification and analysis of RNA editing

Mismatches in the RNA-Seq reads were first examined to ensure the overall quality of the mismatch calls^32^. This step removes likely sequencing errors based on base call quality, mismatch nucleotide changes and mismatch position in the reads. We then filtered these mismatches by removing those located in homopolymers, splice sites, simple repeats, and those whose read coverage demonstrated a strand bias^33^. These sites were further processed using GIREMI^35^ to obtain high-confidence editing sites. GIREMI identifies editing sites based on the mutual information between editing sites and/or SNPs. Since the RNA-seq experiments were conducted in multiple batches, we designed a scheme to reduce the potential batch effects. Specifically, within each batch, multiple RBP knockdown experiments and one control shRNA experiment (2 replicates each) were carried out. We assume that only a minority of RBPs, if any, in a batch regulates the editing of a particular site. Based on this assumption, for each editing site identified in any dataset of a batch, we defined the control editing level as the average of its editing level in all RBP knockdown and control experiments in the same batch. This procedure was omitted for a small number of batches where only one RBP was included. Based on clustering results (Supplementary Fig. 5, Supplementary Fig. 6), this method effectively removed batch effects.

To identify differentially edited sites upon knockdown of an RBP, the editing level of each editing site was compared to the above averaged editing level in the same batch. Since each knockdown experiment had two biological replicates, we estimated the expected variance in the editing level from the two replicates for each editing site using a method similar as in the BEAPR package^56^. Significant differentially edited sites were identified using a normal distribution parameterized by the mean editing level between two replicates and the expected variance calculated above. The FDR was calculated using the Benjamini-Hochberg method^57^. Differentially edited sites were called by requiring FDR ≤ 10% and the absolute change in the editing level between knockdown and control ≥5%. The code for the identification of differentially edited sites is available at https://github.com/gxiaolab/RNA_editing/tree/master/RBP_regulation, together with all differentially edited sites identified in this study.

### Overlap scores of differential editing of two RBPs

We calculated overlap scores to represent the degrees of overlap among differentially edited sites associated with a pair of RBPs. For each pair of RBPs, two overlap scores were defined, represented by two links in the CIRCOS plots (Fig. 1a). The scores correspond to the thickness and color of the links in the plots. To calculate these scores, we first obtained the number of shared differentially edited sites of two RBPs. The numbers of differentially edited sites with the same (*n*_1_) or opposite (*n*_2_) directions in their changes of editing levels upon knockdown were obtained. Pairs of RBPs with less than 20 total shared differentially edited sites were not considered (thus with no links in the plot). Then, we obtained the number (*t*) of shared testable sites in the datasets of the two RBPs. The ratios *n*_1_/*t* and *n*_2_/*t* were then calculated, where *n*_2_/*t* was reported as -*n*_2_/*t* to represent the opposite directions in editing changes. The final overlap scores are defined as the Z-score of these ratios across all RBP pairs for each cell line.

### Global direction of editing regulation

We tested whether there exists a significant bias in the direction of editing changes (higher or lower relative to controls) caused by the knockdown of an RBP using a bootstrap sampling approach (Figs. 2, 3, and 5). For each RBP-knockdown sample, we obtained the total number of differentially edited sites (*n*) and the fraction of these differentially edited sites with increased editing level upon knockdown (*r*). We then randomly sampled *n* sites from all testable sites of the same RBP-knockdown dataset and calculated a similar fraction $(r_i^ \ast )$. We repeated this random sampling process 100,000 times to obtain an empirical distribution of the ratios: $r^ \ast = r_1^ \ast ,r_2^ \ast , \ldots ,r_{100,000}^ \ast$. The *z*-score of *r* was therefore defined as $z = \frac{{r - \widehat {r^ \ast }}}{{\sigma _{r^ \ast }}}$ where $\widehat {r^ \ast }$ and $\sigma _{r^\ast }$ were the mean and standard deviation of r*, respectively. Finally, the empirical *p* value of *r* was calculated by comparing to r*.

For TARDBP (Fig. 2c), we additionally tested the significance of change in the global editing levels for all testable sites. We performed a similar test as described above, but by randomly sampling sites from all testable sites of all RBP knockdown datasets in the same batch as TARDBP.

### WGCNA clustering

To examine whether subsets of RBPs function similarly in regulating RNA editing, we carried out a clustering analysis of RBPs using the Weighted Gene Co-expression Network Analysis^58^. This method finds networks (modules) of nodes based on their topological overlap. For each cell line, the nodes of the WGNCA network consisted of all the RBPs with knockdown data. The edge scores between the nodes (i.e., RBPs) were calculated using pairwise correlation (bicorrelation as recommended by WGCNA) between their differential editing levels between knockdown and controls. We employed WGCNA to create signed networks, which required a soft threshold of 12 to satisfy scale-free topology^36^. Modules in the resulting dendrograms were then examined manually (Supplementary Fig. 6).

### eCLIP-seq analysis

eCLIP-seq data of 126 and 109 RBPs in K562 and HepG2 cells, respectively, were adapter-trimmed and de-multiplexed^59^. For each RBP, we obtained eCLIP-seq data from two biological replicates and one size-matched input control^59^.

To accommodate potential Alu-binding proteins whose eCLIP reads may not align uniquely, the eCLIP data were analyzed using a step-wise mapping procedure^38^. Specifically, the reads were aligned to rRNA sequences first. This step helps to control for spurious artifacts possibly caused by reads derived from rRNA. Those that did not align to rRNAs were retained and aligned to the Alu sequences located in RefSeq genes. This step allows up to 100 multiple alignments per read, maximizing the number of reads that map to Alu elements. Subsequently, reads that did not map to Alu sequences were aligned to the human genome (hg19), where only uniquely-mapped reads were retained. All the alignments were performed by the STAR aligner^60^ with ENCODE standard parameters (as specified in the STAR manual). All alignments were required to be end-to-end without soft-clipping. eCLIP peaks were called using a Poisson model^38^ by requiring a Bonferroni-corrected *p* value cutoff of 0.01.

Next, we examined whether the distance between differentially edited sites upon an RBP knockdown and the eCLIP peaks of the RBP is significantly closer than expected by chance. For each differentially edited site, we calculated its distance to the closest eCLIP peak within the same gene. differentially edited sites in genes that do not have an eCLIP peak were discarded. As control sites, we used known editing sites from the REDIportal database^7^ that satisfy the following: ≥15 combined total read coverage from the two replicates; located in the same gene as the differentially edited site; not identified with edited reads in any dataset of our study. For ADAR1, we used non-REDIportal A’s as random controls, instead of known editing sites.

For each RBP, we randomly selected the same number of control sites as that of differentially edited sites and calculated the distance between a control site and the closet eCLIP peak within the same gene. We repeated this process 200 times to generate 200 sets of controls. The distances of each set of controls to eCLIP peaks were visualized via empirical cumulated distribution function (eCDF), similarly for the distances of the actual differentially edited sites to eCLIP peaks. Next, we calculated the area under the curve (AUC) of each distance eCDF and compared the AUC corresponding to differentially edited sites and those resulted from the 200 sets of control sites. It is expected that smaller distances lead to larger AUCs. Thus, to determine whether the differentially edited sites were significantly closer to eCLIP peaks than expected by chance, we calculated the two-sided p-value by fitting the AUC values of the controls with a normal distribution. In addition, a fold-change was calculated as the ratio between the AUC associated with differentially edited sites and the mean AUC of the control eCDFs.

### Directional agreement score

For each pair of RBPs tested in the same cell line, we took the union of their associated differentially edited sites and further retained only those sites that are testable in both RBP knockdown datasets. Testable sites were defined as those with ≥5 total reads per replicate, and with ≥10% editing level in either knockdown or control. Using these editing sites, we asked whether the directions of editing changes upon knockdown of the two RBPs are concordant by calculating the directional agreement score. Specifically, for each of the above differentially edited site, we labeled it as + or − if its change in editing level upon RBP knockdown is a positive or a negative value, respectively. For sites with the same label for both RBPs, a +1 agreement score was assigned. Otherwise, a score of −1 was given. If the editing site is differentially edited in only one of the two RBP knockdowns, a score 0 was given. The final directional agreement score of a pair of RBPs is defined as the average value of the score of each included editing site in this analysis.

The directional agreement score of the same RBP between K562 and HepG2 was calculated similarly.

### Co-immunoprecipitation

Cells were maintained with DMEM supplemented with 10% FBS and 100 U mL^−1^ penicillin/streptomycin at 37 °C and 5% CO_2_. Ten million cells were collected and lysed in 1 mL non-denaturing lysis buffer at pH 8.0, containing 20 mM Tris-HCl, 125 mM NaCl, 1% NP-40, and 2 mM EDTA supplemented with complete protease inhibitor cocktail. Extracted proteins were incubated overnight with DROSHA antibody (Bethyl Laboratories, A301-886A) at 4 °C; precipitation of the immune complexes was performed with Dynabeads Protein G (Thermo Fisher Scientific, 1003D) for 4 h at 4 °C, according to the manufacturer’s instructions. After immunoprecipitation, the beads were washed three times with the lysis buffer at 4 °C and eluted from the Dynabeads using elute buffer (0.2 M glycine, at pH 2.8). Twenty microliters were loaded onto the gel and the samples were processed by SDS-polyacrylamide gel electrophoresis (SDS-PAGE) and analyzed by Western blot. The following antibodies were used for the Western blots: ADAR1 antibody (Santa Cruz, sc-73408) and DROSHA antibody (Bethyl Laboratories, A301-886A). The HRP-linked secondary antibodies were used and the blots were visualized with the ECL kit (GE, RPN2232).

### Constructs, transfection, luciferase reporter assay

TDP-43 ChIP peak regions were cloned into a firefly luciferase reporter pGL3 vectors (Promega). The pSV40-Renilla vector (Promega) encoding the Renilla luciferase reporter gene Rluc (*Renilla reniformis*) was used for transfection efficiency. Transfections were performed with the use of Lipofectamine 3000 (Invitrogen). HepG2 cells were seeded into 12 well plates at a density of 2.0 × 10^5^ cells per well the day before transfection. For each well of cells 1.0 μg of the pGL3 constructs were co-transfected with 0.1 μg of the pSV40-Renilla vectors. The transfected cells were collected after 48 h. Luciferase activities were measured with the Dual-Luciferase Reporter Assay System (Promega, E1910). To normalize for transfection efficiency, the reporter activity was expressed as the ratio of firefly activity to renilla activity. For each construct, three independent experiments were performed in triplicate.

### Code availability

Scripts for differential editing analysis (and related results) are available at https://github.com/gxiaolab/RNA_editing/tree/master/RBP_regulation.

## Supplementary information

Supplementary Information

Supplementary Data 1

Description of Additional Supplementary Files

## Data Availability

All data sets used in this study can be obtained from the ENCODE project website at http://www.encodeproject.org. We used shRNA RNA-Seq and eCLIP-Seq datasets in HepG2 and K562 cells with release dates between October 2014 and January 2017. The data underlying the main figures are available in Supplementary Data 1.
