# Supplementary material for: Regulation of RNA editing by RNA-binding proteins in human cells
Source: Commun Biol. 2019 Jan 14;2:19. doi: 10.1038/s42003-018-0271-8 (PMC6331435; doi:10.1038/s42003-018-0271-8)
Supplement: Supplementary file 1 — Supplementary Information [file 42003_2018_271_MOESM1_ESM.pdf]

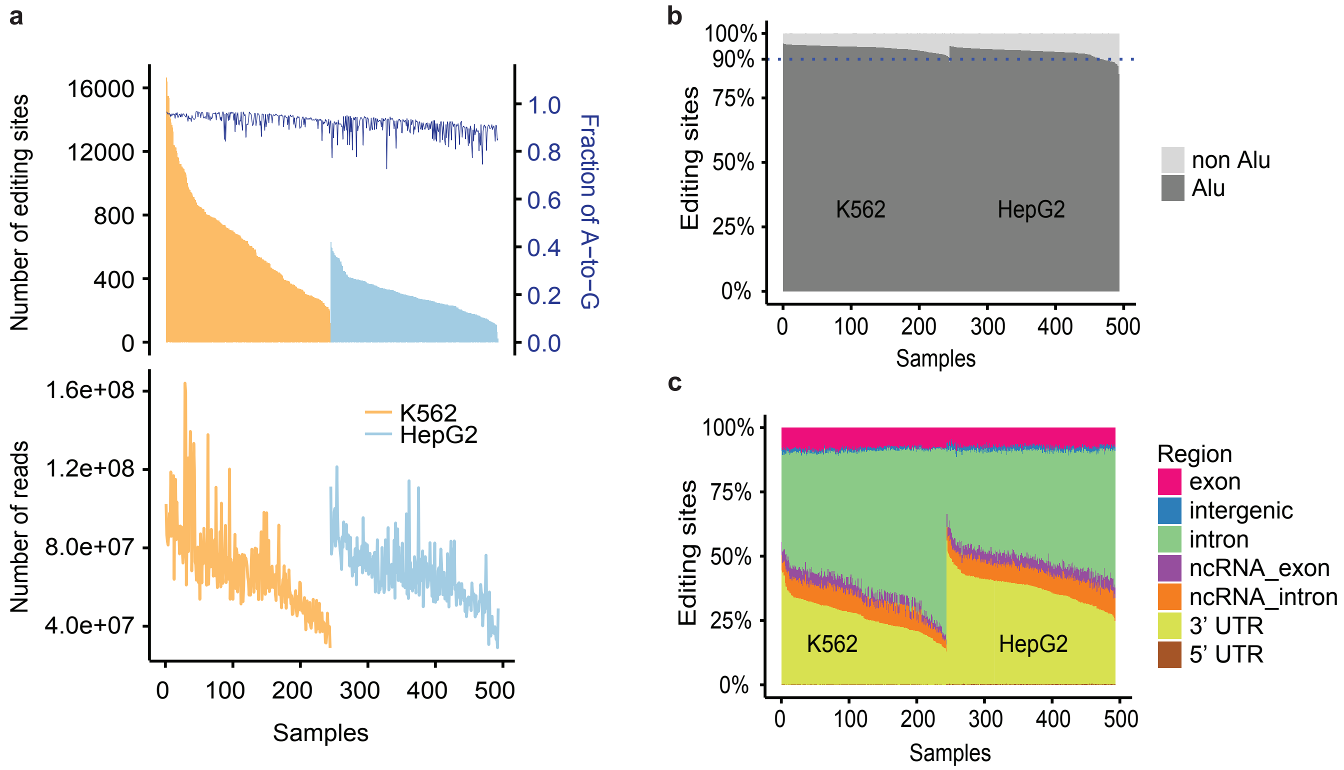


**Supplementary Figure 1**. (a) Number of testable editing sites (top) and total RNA-Sequencing depth per samples in K562 and HepG2 cells. Fraction of A-to-G editing sites also shown for each sample (top, blue line). (b) Percentage of editing sites in Alu regions for each sample. (c) Genomic distribution of editing sites. ncRNA refers to non-coding transcripts.

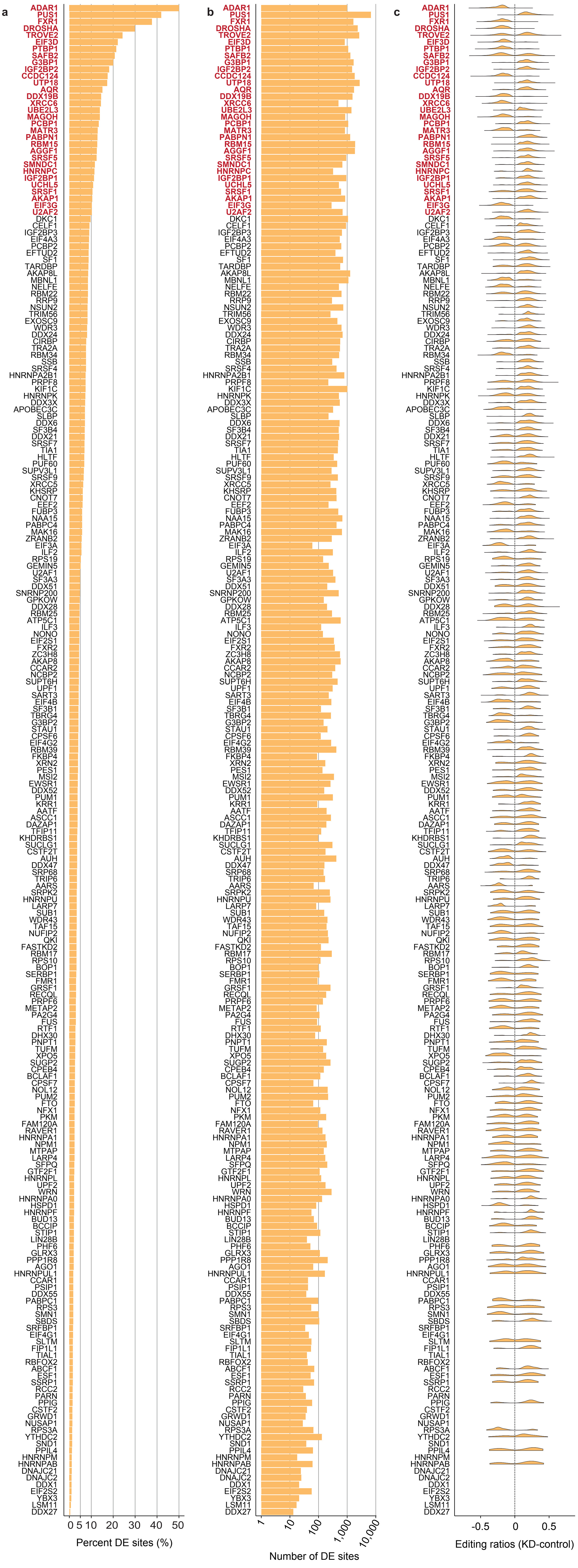


**Supplementary Figure 2 (a-c)**. Differential editing associated with RBPs in K562 cells. (a) Percentage of differentially edited (DE) sites among all testable sites associated with each RBP in HepG2 cells. (b) Number of DE sites associated with each RBP. (c) Distribution of editing changes of all DE sites associated with each RBP. Only RBPs with more than 50 DE sites are shown. RBPs whose DE sites comprise at least 10% of all testable editing sites are highlighted in red.

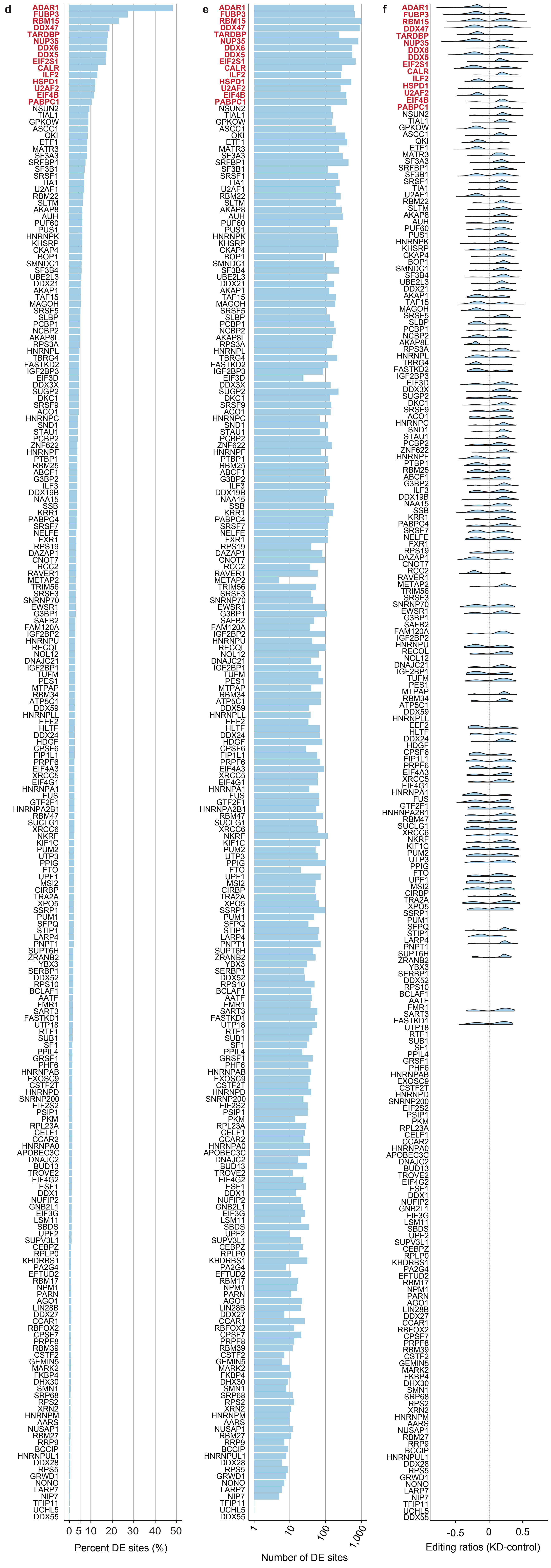


**Supplementary Figure 2 (d-f)**. Differential editing associated with RBPs in HepG2 cells. (d) Percentage of differentially edited (DE) sites among all testable sites associated with each RBP in HepG2 cells. (e) Number of DE sites associated with each RBP. (f) Distribution of editing changes of all DE sites associated with each RBP. Only RBPs with more than 50 DE sites are shown. RBPs whose DE sites comprise at least 10% of all testable editing sites are highlighted in red.

**a**

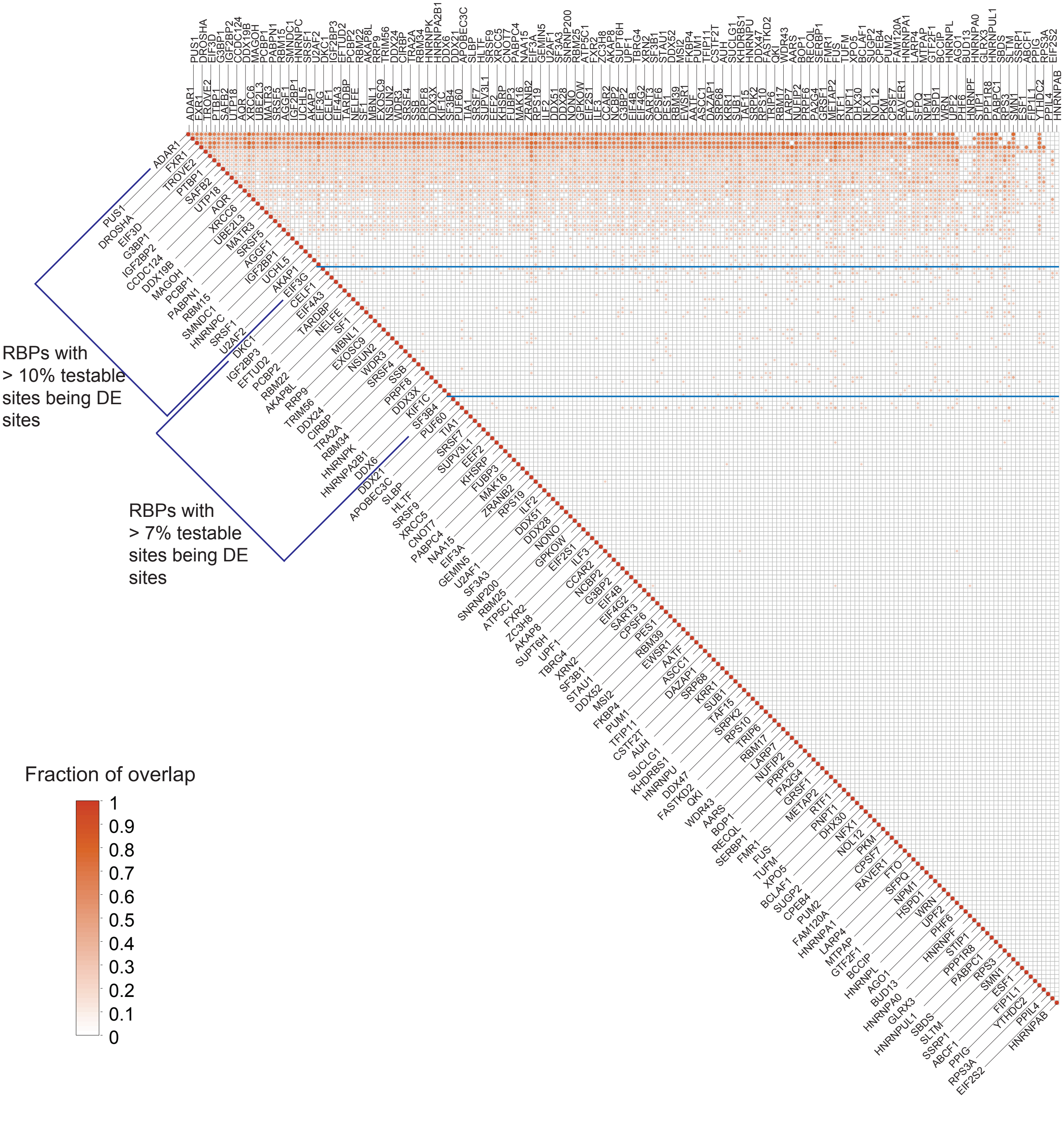


**Supplementary Figure 3**. (a) Overlap of differentially edited (DE) sites between pairs of RBP knockdown samples in K562 cells. The fraction of overlap was calculated as the number of common differentially edited sites over the minimum number of differentially edited sites sites between the two samples. Values < 0.25 were not shown for visualization purpose. RBPs are sorted from top to bottom based on their average fraction of overlap with all other RBPs (RBPs at the top have the highest values). RBPs whose differentially edited sites comprise >10% or 7% of all testable sites are highlighted.

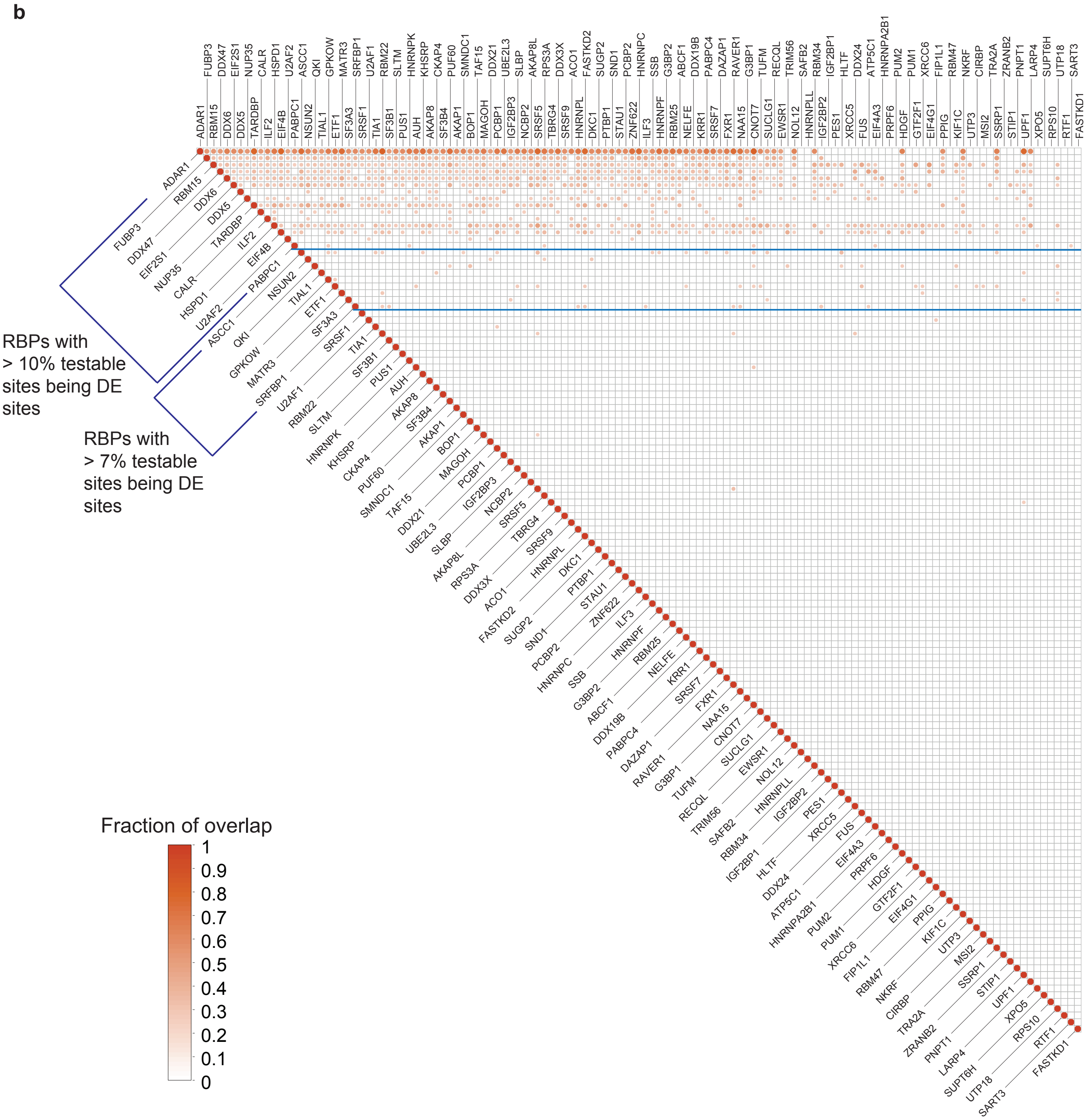


**Supplementary Figure 3**. (b) Overlap of differentially edited sites between pairs of RBP knockdown samples in HepG2 cells. The fraction of overlap was calculated as the number of common differentially edited (DE) sites over the minimum number of differentially edited sites between the two samples. Values < 0.25 were not shown for visualization purpose. RBPs are sorted from top to bottom based on their average fraction of overlap with all other RBPs (RBPs at the top have the highest values). RBPs whose differentially edited sites comprise >10% or 7% of all testable sites are highlighted.

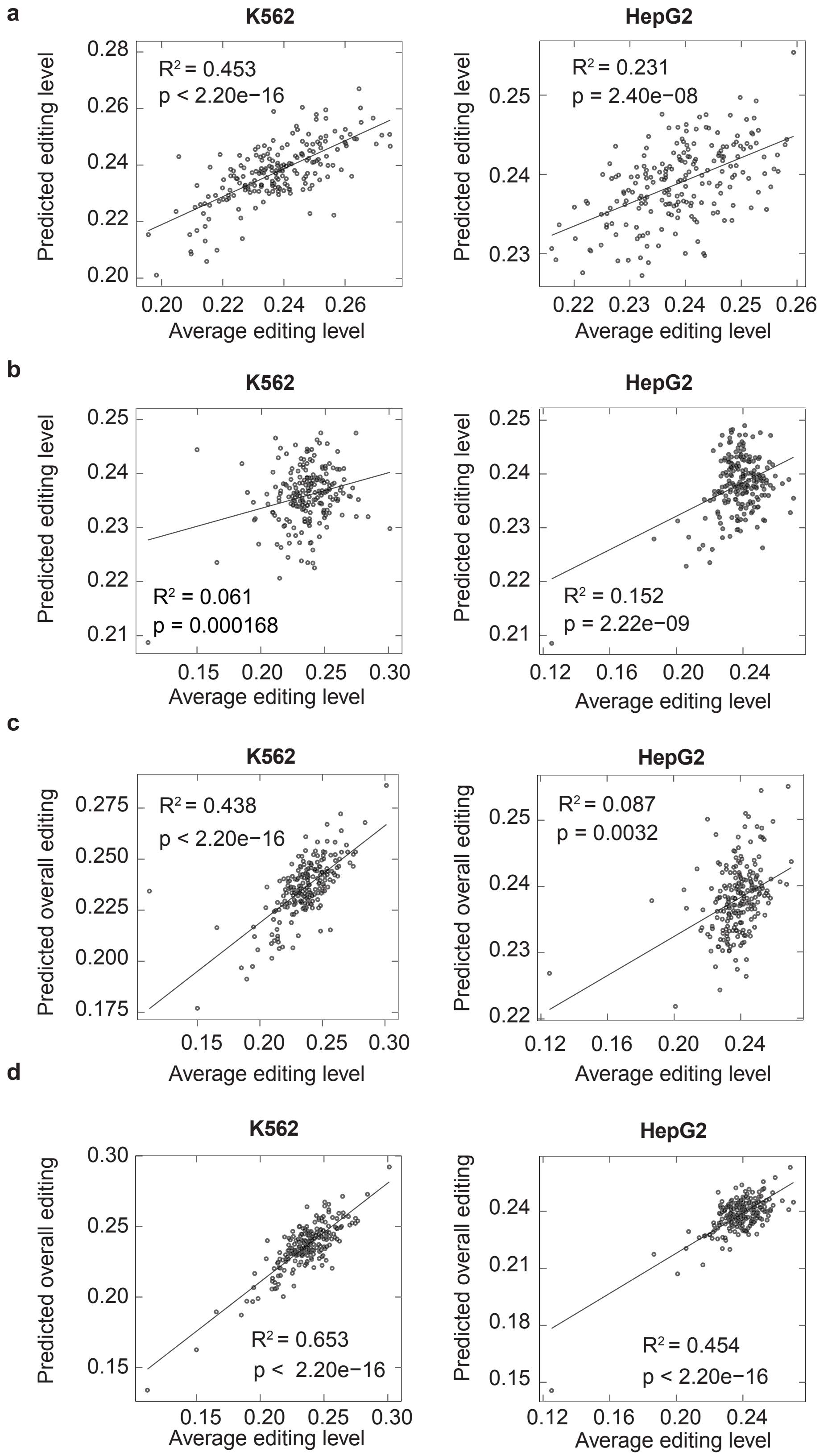


**Supplementary Figure 4.** Regression analysis between the observed average editing levels in each RNA-Seq dataset and the predicted editing level (Similar to Fig. 1c). The predicted editing level is based on: (a) the mRNA expression of the top 15 RBPs (including ADAR1) with greatest impact on editing regulation (the top 10 highest leverage points were removed to test their influence on the regression). (b) the mRNA expression of ADAR1 alone. (c) the expression of the remaining 14 RBPs (excluding ADAR1). (d) the expression of the top 15 RBPs (including ADAR1) and ADAR1-RBP interaction terms.

a

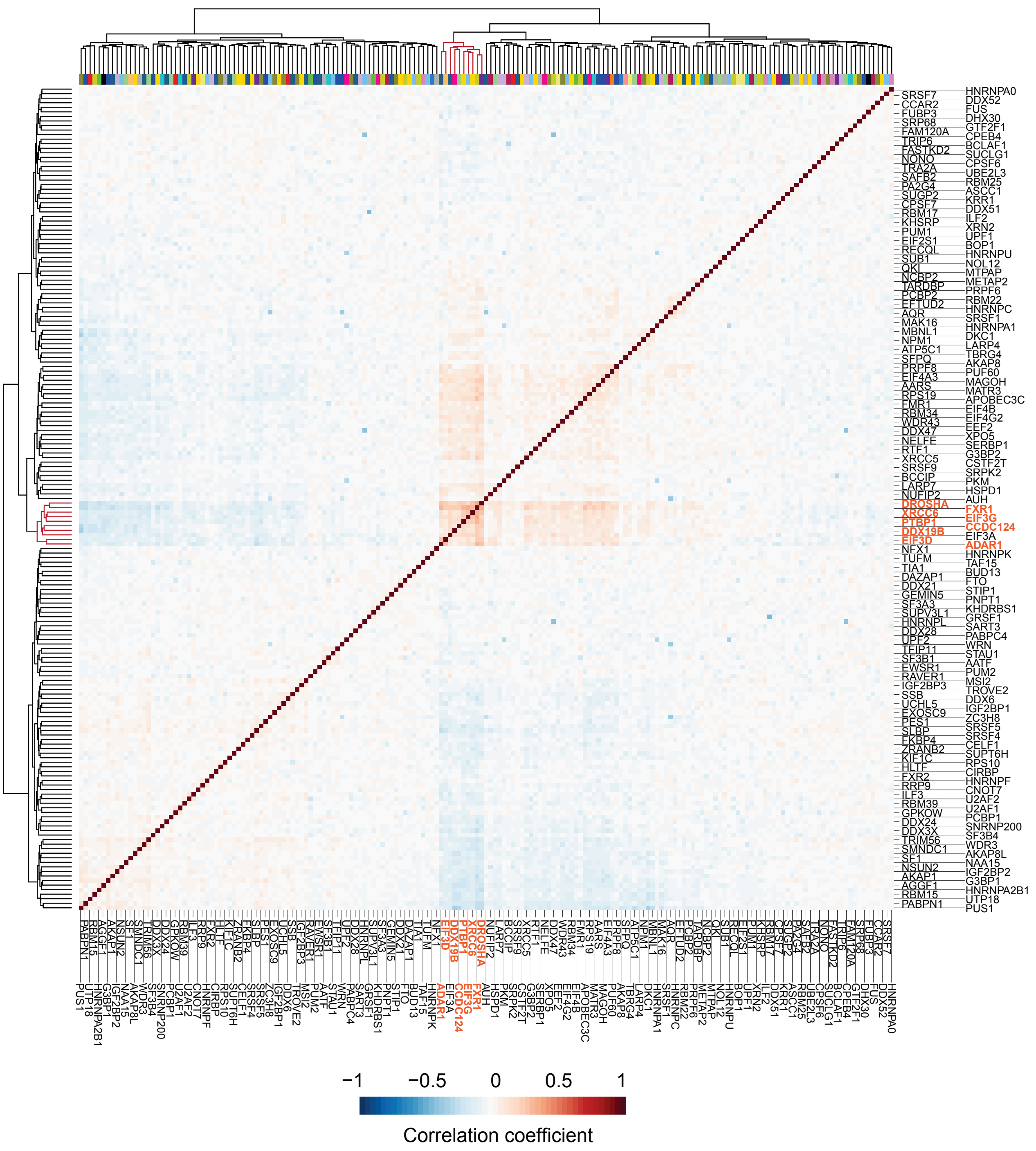


**Supplementary Figure 5**. (a) Hierarchical clustering of pair-wise Spearman correlation of editing changes upon RBP knockdown in K562 cells. The union of all differentially edited sites identified in the K562 knockdown samples is used. For each pair of RBPs, only differentially edited sites that are testable in both datasets are included. The small cluster shown in red is associated with the highest correlation coefficients. This cluster contains RBPs associated with most significant reduction in editing (based on percentage of differentially edited sites among all testable sites) upon their knockdown. RBPs are labeled in orange in this cluster if they are associated with >10% differentially edited among all testable sites. The color labels on top denote experimental batches of each RBP.

**b**

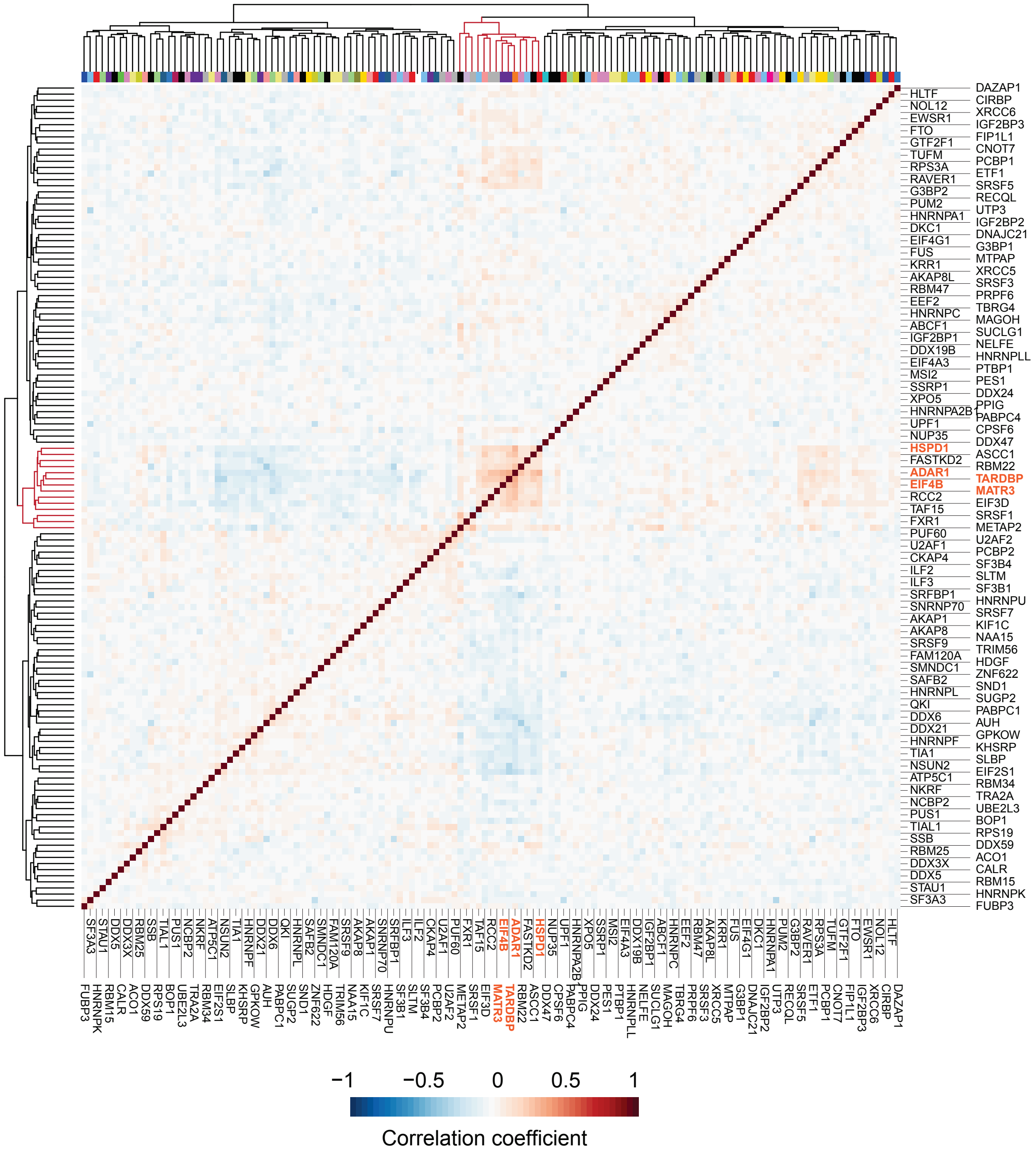


**Supplementary Figure 5**. (b) Hierarchical clustering of pair-wise Spearman correlation of editing changes upon RBP knockdown in HepG2 cells. The union of all differentially edited sites identified in the HepG2 knockdown samples is used. For each pair of RBPs, only differentially edited sites that are testable in both datasets are included. The small cluster shown in red is associated with the highest correlation coefficients. This cluster contains RBPs associated with most significant reduction in editing (based on percentage of differentially edited sites among all testable sites) upon their knockdown. RBPs are labeled in orange in this cluster if they are associated with >10% differentially edited among all testable sites. The color labels on top denote experimental batches of each RBP.

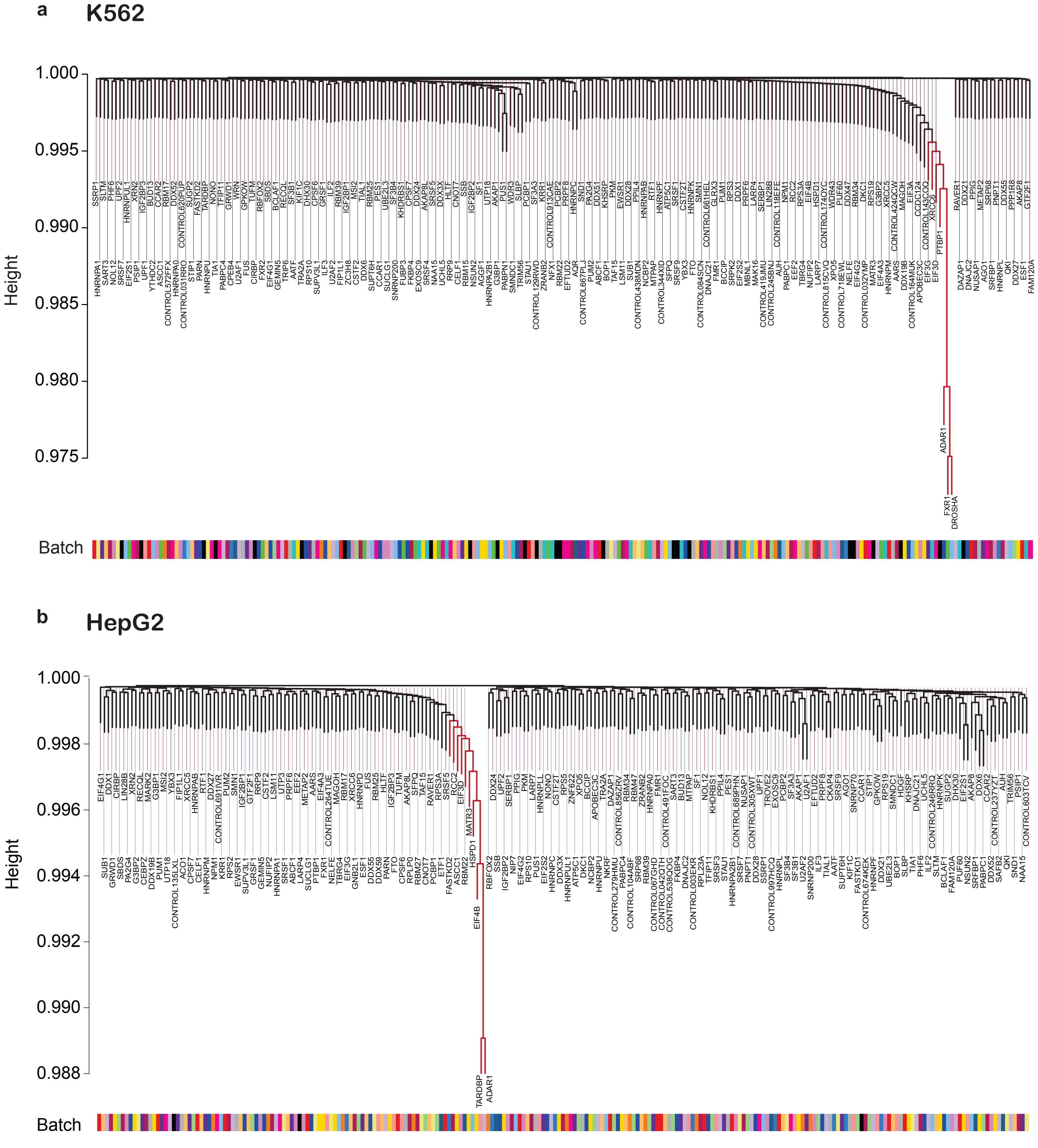


**Supplementary Figure 6.** WGCNA clustering (see Methods) of RBP knockdown data and controls. Correlation between RBPs were calculated via biweight mid-correlation using the change in editing levels upon RBP knockdown. The clusters in red contain RBPs with high percentage of differentially edited sites among all testable sites. These RBPs are associated with reduction in editing upon their knockdown. (a) K562 (b) HepG2. The color labels at the bottom denote experimental batches of each RBP.

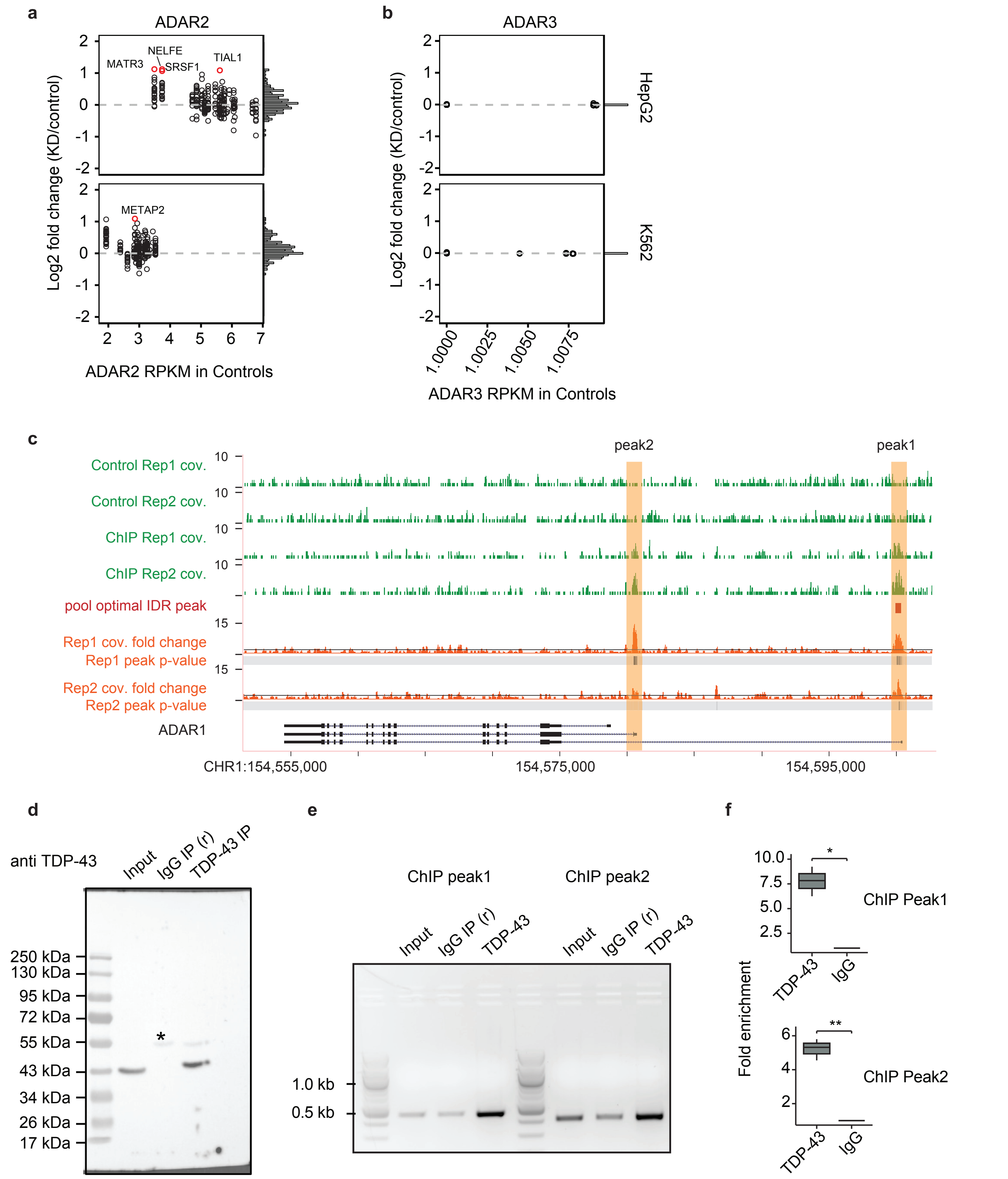


**Supplementary Figure 7.** mRNA expression (RPKM) of (a) ADAR2. (b) ADAR3. Log2 fold changes (LFCs) of expression levels (knockdown/control) are shown. RBPs whose knockdown induced LFC > 1 and DESeq p value < 1e-9 are labeled in red. Histograms of LFC values are shown on the side of the plots. RPKM values have a pseudocount of 1. (c) Genome Browser view of TDP-43 ChIP-Seq datasets. In green, the read coverage of the ChIP samples and controls. In orange, the coverage fold change (ChIP vs control) of the two replicates. A line is drawn at fold change = 2. The peak coordinates and p-values were obtained using *spp* peak caller by the ENCODE consortium. P-values smaller than 0.01 are shown in dark gray tones. The peak (peak1) that passed the optimal irreproducible discovery rate (IDR) criteria of ENCODE is illustrated in the track named “pool optimal IDR peak” in red. (d) Western Blot of TDP-43 Immuno precipitation in HepG2 cells. The asterisk marks cross reaction with the antibody heavy chain. (e) Semi-quantitative PCR products from TDP-43 ChIP, input control and IgG using primers targeting peak1 and peak2 respectively. (f) Real-time qPCR of products from TDP-43 ChIP and IgG (BioRad qPCR Analysis Software, CFX Maestro Software). The values corresponds to the fold enrichment of the band intensity (IP/input). Error bars represent standard deviation calculated from 3 biological replicates (Student’s t-test, *P<0.05, **P<0.01).

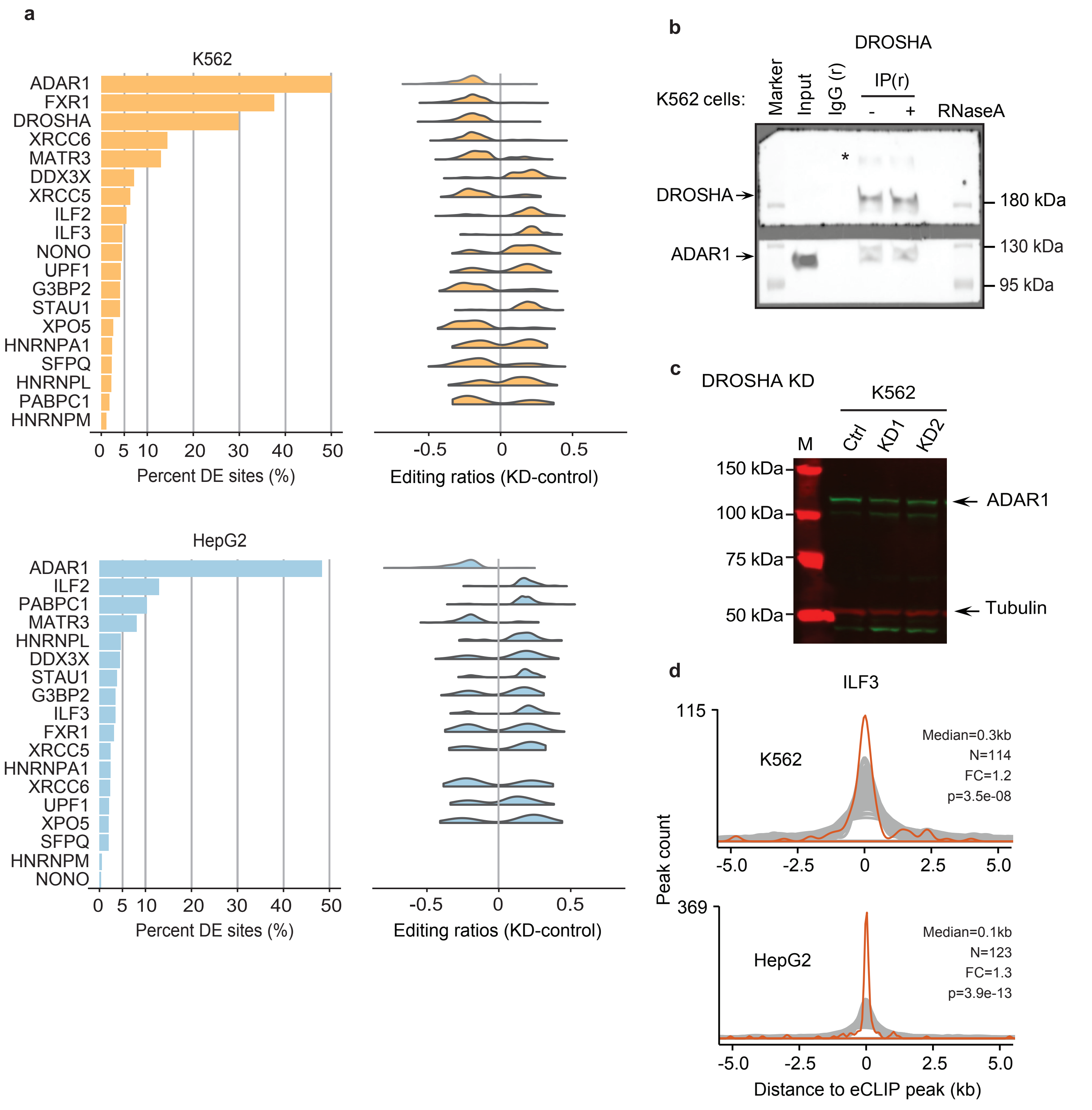


**Supplementary Figure 8.** (a) RNA editing regulation by ADAR1-interacting RBPs. RBPs with shRNA RNA-Seq data from ENCODE known to interact with ADAR1 in the literature were selected. Left: percentages of testable editing sites that are differentially edited (DE) upon RBP knockdown (KD) are shown. Right: distributions of editing changes of the differentially edited sites associated with each RBP are shown (except for RBPs with less than 50 differentially edited sites). (b) Uncropped blot image of the DROSHA and ADAR1 Co-Immunoprecipitation from Fig. 3c. The asterisk indicates non-specific bands. The gel was cut to use for multiple antibodies. The image in Fig. 3c is a re-exposed version of this gel. (c) Western blot of shRNA-mediated DROSHA knockdown. Both K562 and HepG2 cells were transduced with lentiviruses expressing pLKO.1-DROSHA shRNA (knockdown) and pLKO.1-control shRNA (control), followed by selection with puromycin to establish stable cell lines. Blots were probed with antibodies detecting ADAR1 and Tubulin control. (d) Distance between ILF3 eCLIP peaks and their closest differentially edited sites (orange). Gray curves represent such distances relative to control sites (see Methods).

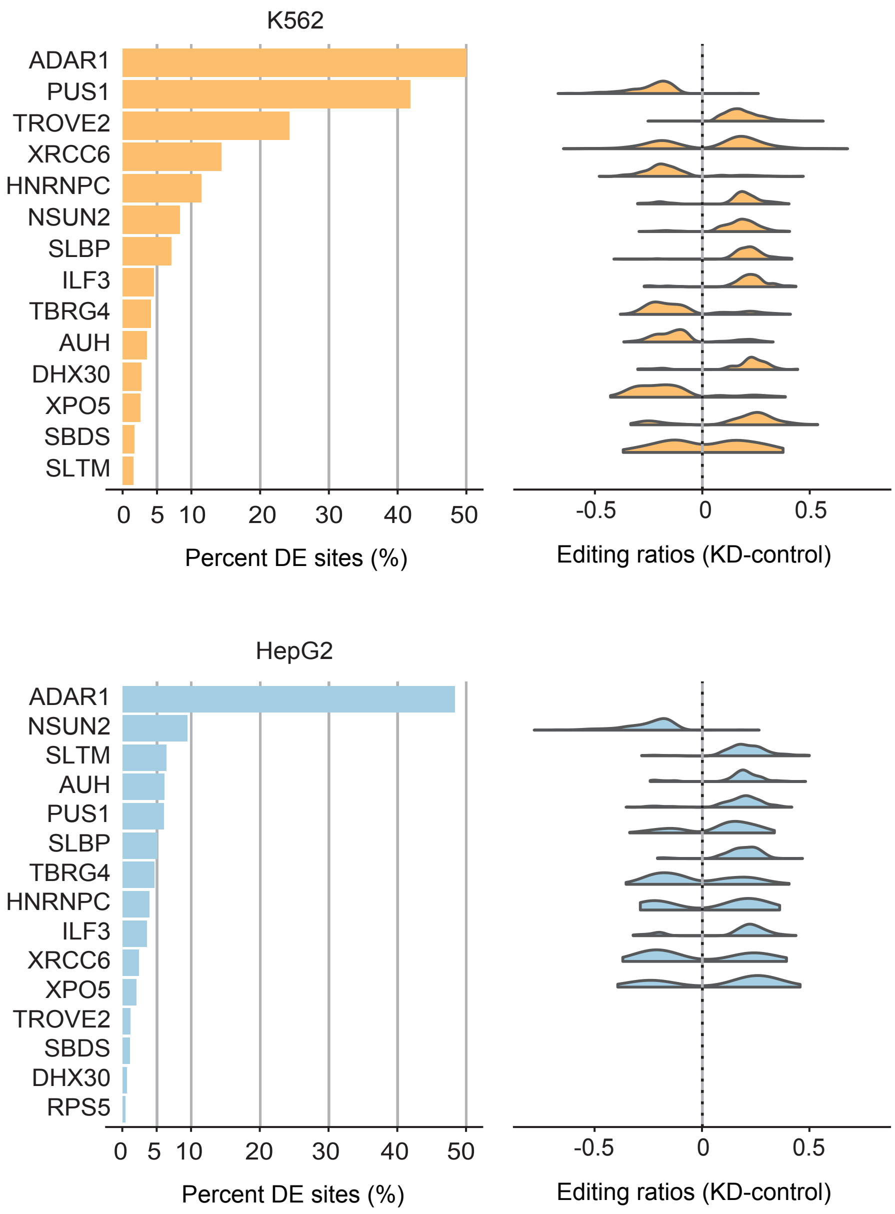


**Supplementary Figure 9.** RNA editing regulation by Alu-binding RBPs. The top 10 RBPs with highest fraction of eCLIP peaks overlapping alu regions for each cell line were selected. Left: percentages of testable editing sites that are differentially edited (DE) upon RBP knockdown (KD) are shown. Right: distributions of editing changes of the differentially edited sites associated with each RBP are shown (except for RBPs with less than 50 differentially edited sites).

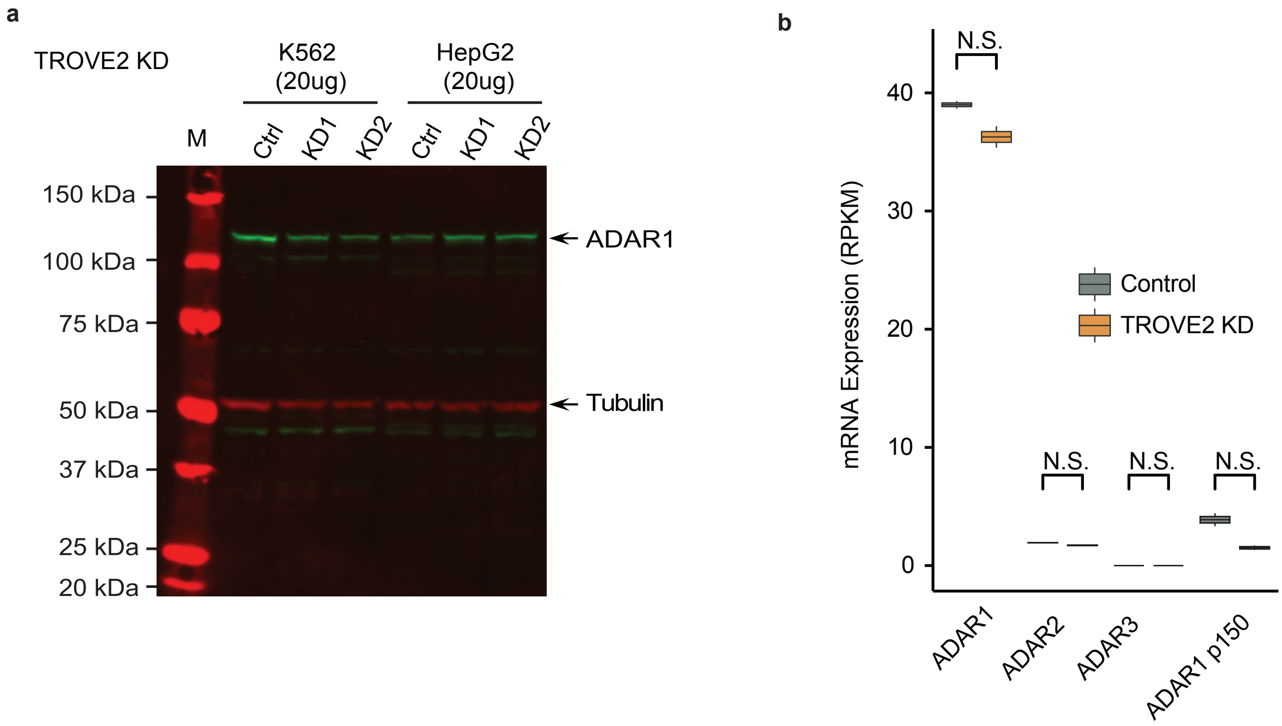


**Supplementary Figure 10**. (a) Western blot of shRNA-mediated TROVE2 knockdown (KD). Both K562 and HepG2 cells were transduced with lentiviruses expressing pLKO.1-TROVE2 shRNA (knockdown) and pLKO.1-control shRNA (control), followed by selection with puromycin to establish stable cell lines. Blots were probed with antibodies detecting ADAR1 and Tubulin (as a control). (b) Expression of ADAR transcripts in TROVE2 knockdown K562 cells (N = 2 biological replicates, P-values were obtained using DESeq).

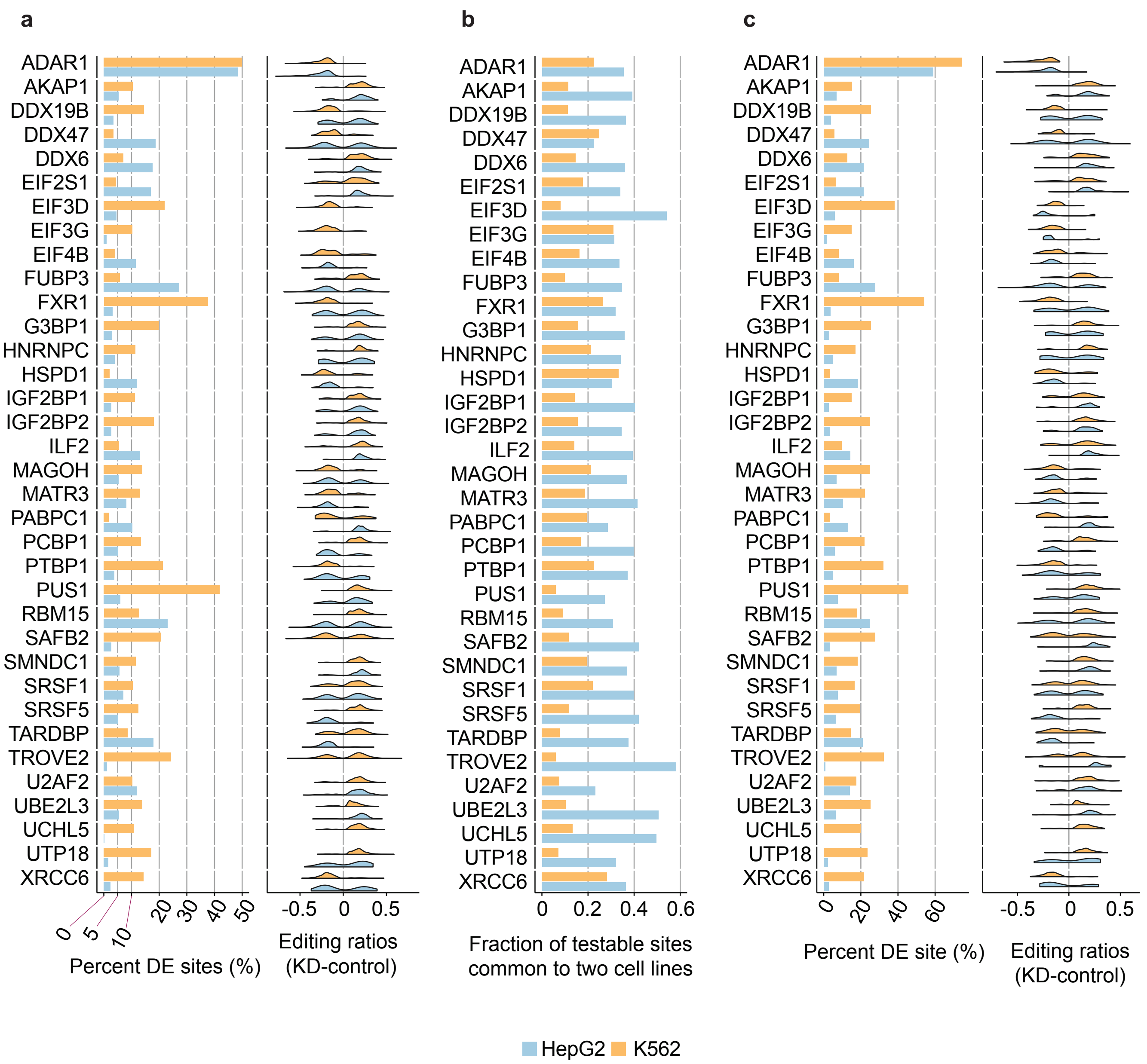


**Supplementary Figure 11.** RBPs with at least 10% of testable sites being differentially edited (DE) upon knockdown (KD) in at least one cell line. (a) Left: percentages of testable editing sites that are differentially edited upon RBP knockdown are shown. Right: distributions of editing changes of the differentially edited sites associated with each RBP are shown (except for RBPs with less than 50 differentially edited sites). (b) Fraction of testable editing sites that are common to both cell lines. (c) Percetage of the common testable editing sites that are differentially edited upon RBP knockdown (left) and editing changes of these sites (right).

**Supplementary Table 1. List of RBPs in this study that are known to interact with ADAR1**

| RBP | Reference | Interaction type | Assay | Cell line |
|---|---|---|---|---|
| DDX3X | 1 | Through complex | Co-IP | 293 |
| DROSHA | 2 | RNA independent | Co-IP | HeLa |
| FXR1 | 3 | RNA independent | Co-IP | HeLa |
| G3BP2 | 4 | | nano-LC MS | 293T |
| HNRNPA1 | 5,6 | | Flag-IP MS, BAC-GFP | Hek293, HeLa |
| HNRNPL | 4 | RNA dependent | nano-LC MS | 293T |
| HNRNPM | 7 | | LC-MS/MS | HeLa |
| ILF2 | 1,8 | RNA dependent | Co-IP | SGC7901 |
| ILF3 | 8,9 | RNA dependent (dsRNA only) | Co-IP | SGC7902 |
| MATR3 | 6 | | BAC-GFP | HeLa |
| NONO | 4 | RNA independent | nano-LC MS | 293T |
| PABPC1 | 4 | RNA dependent | nano-LC MS | 293T |
| SFPQ | 4 | RNA dependent | nano-LC MS | 293T |
| STAU1 | 10 | RNA dependent | Co-IP | Hek293T |
| UPF1 | 11 | RNA independent | Co-IP | HeLa |
| XPO5 | 12 | RNA independent | Co-IP | HeLa |
| XRCC5 | 13 | Through complex | Co-IP | Hek293T |
| XRCC6 | 13 | Through complex | Co-IP | Hek293T |

## Supplementary References


1. Guan, D. *et al.* Nuclear Factor 45 (NF45) Is a Regulatory Subunit of Complexes with NF90/110 Involved in Mitotic Control. *Mol. Cell. Biol.* **28,** 4629–4641 (2008).
2. Bahn, J. H. *et al.* Genomic analysis of ADAR1 binding and its involvement in multiple RNA processing pathways. *Nat. Commun.* **6,** 6355 (2015).
3. Tran, S. *et al.* Widespread RNA editing dysregulation in Autism Spectrum Disorders. *Under review.*
4. Orecchini, E. *et al.* ADAR1 restricts LINE-1 retrotransposition. *Nucleic Acids Res.* **45,** 155–168 (2017).
5. Close, P. *et al.* DBIRD complex integrates alternative mRNA splicing with RNA polymerase II transcript elongation. *Nature* **484,** 386–389 (2012).

6. Hein, M. Y. *et al.* A Human Interactome in Three Quantitative Dimensions Organized by Stoichiometries and Abundances. *Cell* **163,** 712–723 (2015).
7. Havugimana, P. C. *et al.* A census of human soluble protein complexes. *Cell* **150,** 1068–1081 (2012).
8. Nie, Y., Ding, L., Kao, P. N., Braun, R. & Yang, J.-H. ADAR1 interacts with NF90 through double-stranded RNA and regulates NF90-mediated gene expression independently of RNA editing. *Mol. Cell. Biol.* **25,** 6956–63 (2005).
9. Wang, I. X. *et al.* ADAR Regulates RNA Editing, Transcript Stability, and Gene Expression. *Cell Rep.* **5,** 849–860 (2013).
10. Elbarbary, R. A., Li, W., Tian, B. & Maquat, L. E. STAU1 binding 3′ UTR IRAlus complements nuclear retention to protect cells from PKR-mediated translational shutdown. *Genes Dev.* **27,** 1495–1510 (2013).
11. Agranat, L., Raitskin, O., Sperling, J. & Sperling, R. The editing enzyme ADAR1 and the mRNA surveillance protein hUpf1 interact in the cell nucleus. *Proc. Natl. Acad. Sci.* **105,** 5028–5033 (2008).
12. Fritz, J. *et al.* RNA-Regulated Interaction of Transportin-1 and Exportin-5 with the Double-Stranded RNA-Binding Domain Regulates Nucleocytoplasmic Shuttling of ADAR1. *Mol. Cell. Biol.* **29,** 1487–1497 (2009).
13. Wang, Q., Zhang, Z., Blackwell, K. & Carmichael, G. G. Vigilins bind to promiscuously A-to-I-edited RNAs and are involved in the formation of heterochromatin. *Curr. Biol.* **15,** 384–391 (2005).
