# Supplementary material for: Regulation of RNA editing by RNA-binding proteins in human cells
Source: Commun Biol. 2019 Jan 14;2:19. doi: 10.1038/s42003-018-0271-8 (PMC6331435; doi:10.1038/s42003-018-0271-8)
Supplement: Supplementary file 3 — Description of Additional Supplementary Files [file 42003_2018_271_MOESM3_ESM.docx]

**Description of Additional Supplementary Files**

**File Name**: Supplementary Data 1

**Description**: Contains the source data underlying each of the main figures. The tabs in this excel file correspond to panels of each figure, as labelled.
